# Tracking Chain Populations and Branching Structure during Polyethylene Deconstruction Processes

**DOI:** 10.1021/acscentsci.4c00951

**Published:** 2024-08-21

**Authors:** Alex H. Balzer, Zachary R. Hinton, Brandon C. Vance, Dionisios G. Vlachos, LaShanda T. J. Korley, Thomas H. Epps

**Affiliations:** † Center for Plastics Innovation (CPI), University of Delaware, Newark, Delaware 19716, United States; ‡ Department of Chemical and Biomolecular Engineering, University of Delaware, Newark, Delaware 19716, United States; § Department of Materials Science and Engineering, University of Delaware, Newark, Delaware 19716, United States; ∥ Center for Research in Soft matter and Polymers (CRiSP), University of Delaware, Newark, Delaware 19716, United States

## Abstract

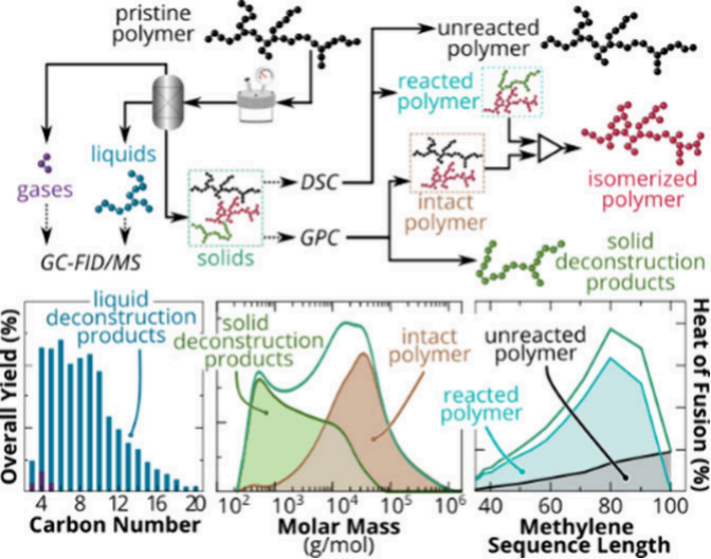

Catalytic deconstruction has emerged as a promising solution to valorize polyethylene (PE) waste into valuable products, such as oils, fuels, surfactants, and lubricants. Unfortunately, commercialization has been hampered by inadequate optimization of PE deconstruction due to an inability to either truly characterize the polymer transformations or adjust catalytic conditions to match the ever-evolving product distribution and associated property changes. To address these challenges, a detailed analysis of molar mass distributions and thermal characterization was developed herein and applied to low-density polyethylene (LDPE) deconstruction to enable more thorough identification of polymer chain characteristics within the solids (e.g., changes in molar mass or branching structure). For example, LDPE hydrocracking exhibited comparable rates of polymer chain isomerization and C–C bond scission, and the solids generated possessed a broadened molar mass distribution with a disappearance of a significant fraction of highly linear segments, indicating polymer-structure-dependent interactions with the catalyst. Solids analysis from pyrolysis yielded starkly different results, as the resulting solids were devoid of unreacted polymer chains and had a narrowed molar mass distribution even at short times (e.g., 0.2 h). By tracking the polymeric deconstruction behavior as a function of reaction type, time, and catalyst design, we mapped critical pathways toward PE valorization.

## Abstract 2

A comprehensive approach to track polymer chain evolution during deconstruction was developed and revealed the influence of deconstruction method and polymer architecture on product distributions.

## Introduction

Low plastics recycling rates (<10%) and the mismanagement of plastics waste have led to the vast majority of plastics directed to landfills.^1−6^ This funneling of waste has produced detrimental ecological impacts, as the majority of consumer plastics do not degrade for several centuries,^7,8^ which motivates significant interest in plastics waste valorization.^9^ Additionally, most plastics are petroleum-derived, and the recovery of carbon from plastics could reduce dependence on oil feedstocks for polymer generation.^10^ Polyethylenes (PEs), which comprise roughly 40 wt % of plastics produced,^11^ can be chemically valorized into carbon feedstocks, such as oils and lubricants.^9,12,13^ Among the major thermochemical valorization techniques (e.g., catalytic hydroconversion,^9,12−16^ pyrolysis,^17−19^ gasification^20−23^), catalytic hydroconversion has shown particular promise as it occurs at milder temperatures and can produce more liquid carbon products, both of which improve economic viability.^24^ The tuning of liquid and gas products traditionally has focused on catalyst and reactor design, and there have been several efforts to decrease costs via earth abundant metal-based catalysts,^25−27^ reduced reaction temperatures,^12,14^ and targeted product yields and selectivity.^28,29^

Interestingly, there has been little emphasis on the molar mass distribution and branching architecture of the PE feedstock or solid products (i.e., solids) and how the evolution of polymeric species affects the conversion.^30^ Similar to how liquid products are affected by the hydroconversion method, the solids properties (e.g., viscosity, thermal conductivity, phase equilibria) are also process-dependent, with the deconstruction mechanism playing a crucial role in shaping these properties. In addition to the molar mass distribution, branching sites along the polymer backbone greatly affect the physical properties,^31^ such as the melt viscosity and chain diffusion of PE, by several orders of magnitude.^32^ From a process and reactor design perspective, as high-molar-mass species transform throughout the reaction, enormous decreases in polymer viscosity and potential phase separation from changes in branching^33−36^ will significantly impact interactions with catalyst particles, thus altering liquid product selectivities.^37^ In the technoeconomic analysis (TEA) and life-cycle assessment (LCA) of PE valorization, solids, if present in the products, are generally treated as being identical to the initial polymer, yet molar mass and branching alterations challenge this assumption.^38^ Given that minimum selling prices for PEs vary by 15–20% and depend on melt-flow properties (i.e., resin type and grade),^39,40^ the quantification of polymer species present in the products allows for more accurate calculations of process viability and may influence the optimized reaction time and conversion. The transformations (e.g., chain scission, isomerization) of polymeric species during hydroconversion and pyrolysis generate solids with physical and thermomechanical properties that are different from the initial solid feedstock, yet a precise metric to predict these changes has not been defined.^41−43^ Overall, deconstruction of PEs is nontrivial and will benefit from understanding the polymer physical properties (e.g., molar mass distributions, branching) that impact this process.

**Figure 1:**
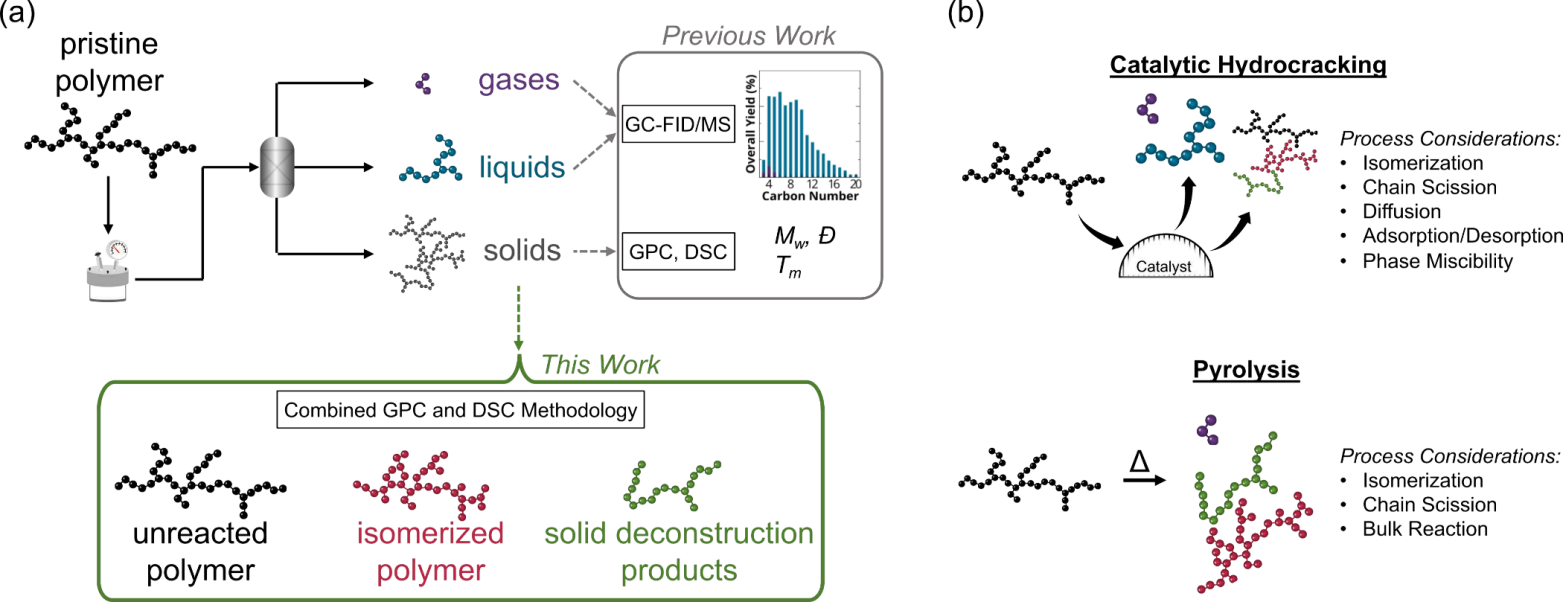
Traditionally, catalytic PE deconstruction processes have been optimized by focusing on the analysis of gas and liquid products through techniques such as gas chromatography with flame-ionization detection and/or mass spectrometry (GC-FID/MS); however, solids in the process have been given limited attention. Solids from deconstruction comprise multiple polymer species, which differ from the pristine feedstock, and their identification is crucial as these species vary across each deconstruction pathway. (a) Herein, a combined and unique GPC and DSC approach is applied to PE deconstruction solids to identify and track the evolution of unreacted, isomerized, and deconstructed polymer chains that expands beyond common macro-level analysis, such as weight-average molar mass (*M*_w_), dispersity (*Đ*), and melting temperature (*T*_m_). (b) The mechanistic differences between catalytic hydrocracking and pyrolysis (via the methods employed in this work) that lead to varying gas and liquid products are shown to produce solids with unique molar mass distributions and branching architectures.

Herein, a comprehensive method that combines molar mass distribution analysis via gel permeation chromatography (GPC) and thermal characterization via differential scanning calorimetry (DSC) is presented that describes the evolution of polymeric species during PE deconstruction via hydrocracking or pyrolysis. The analysis was validated with surrogate PE deconstruction blends of known composition and applied to solids from the deconstruction of low-density polyethylene (LDPE). Mathematical modeling of molar mass distributions from GPC was used to decouple polymer products (or intermediates) from intact polymer (i.e., unreacted feedstock and reacted polymers that have not undergone a measurable change in molar mass). Thermal fractionation via DSC enabled further deconvolution of unreacted polymer from reacted products, and as a result, a more precise accounting of species in the reaction mixture was achieved (Figure 1). To emphasize the utility of this approach, the hydrocracking of LDPE over a platinum tungstated zirconia catalyst was examined, and it was uncovered that as the reaction progressed, the solids were composed of unreacted polymer chains with the relative weight fractions of unreacted and transformed chains dependent on reaction time and metal-acid site balance (MAB) of the catalyst. The pyrolysis of LDPE also was studied, and by contrast, no unreacted chains existed within the solids, and a narrowing of the molar mass distribution occurred, a distinct result that showcases the mechanistic differences found via solids characterization between heterogeneous (hydrocracking) and bulk (pyrolysis) deconstruction. Overall, this report highlights an approach to fully quantify the solid products of catalytic deconstruction to enable the prediction of polymer physical property evolution throughout the reaction and how those changes influence the product species distributions.

## Experimental Section

### System Design and Considerations

At room temperature, reaction mixtures from deconstruction via hydroconversion are composed of solids, liquids, and gases, with liquid alkanes representing the most targeted and valuable products. The characterization of the complex mixtures generated by hydroconversion involves separating the various phases and analyzing their constituent species via gas chromatography and/or mass spectroscopy techniques, with liquid and gas products amenable to precise quantification of carbon lengths and branching.^43,44^ However, the polymeric solid products from hydroconversion are not readily separable, and it is challenging to distinguish between unreacted and reacted chains, when only one or two averaged values (e.g., number/weight-average molar mass from GPC, peak melting temperature from DSC) are considered, versus analyzing the entire distribution and its associated changes. To gain further insight into the evolution of macromolecules into isomerized chains and new chain lengths, and how polymer chain structure affects its interactions with the deconstruction catalyst, it is essential to elucidate the reaction pathways and identify the solid species present.

In catalytic hydrocracking and pyrolysis, solids may possess different chain lengths and architectures relative to the pristine feedstock; therefore, the solids should be appropriately catalogued to account for all species present: unreacted polymer, isomerized polymer, and solid deconstruction products. Unreacted polymer contains the chains that have not changed molar mass or branching architecture (i.e., not reacted with catalyst). Isomerized polymer includes chains with a different branching architecture but the same molar mass as the initial chain. Solid deconstruction products are species that have undergone a change in molar mass and may have been isomerized but are solid at room temperature.

### Surrogate Blends for Methodology Validation

**Figure 2:**
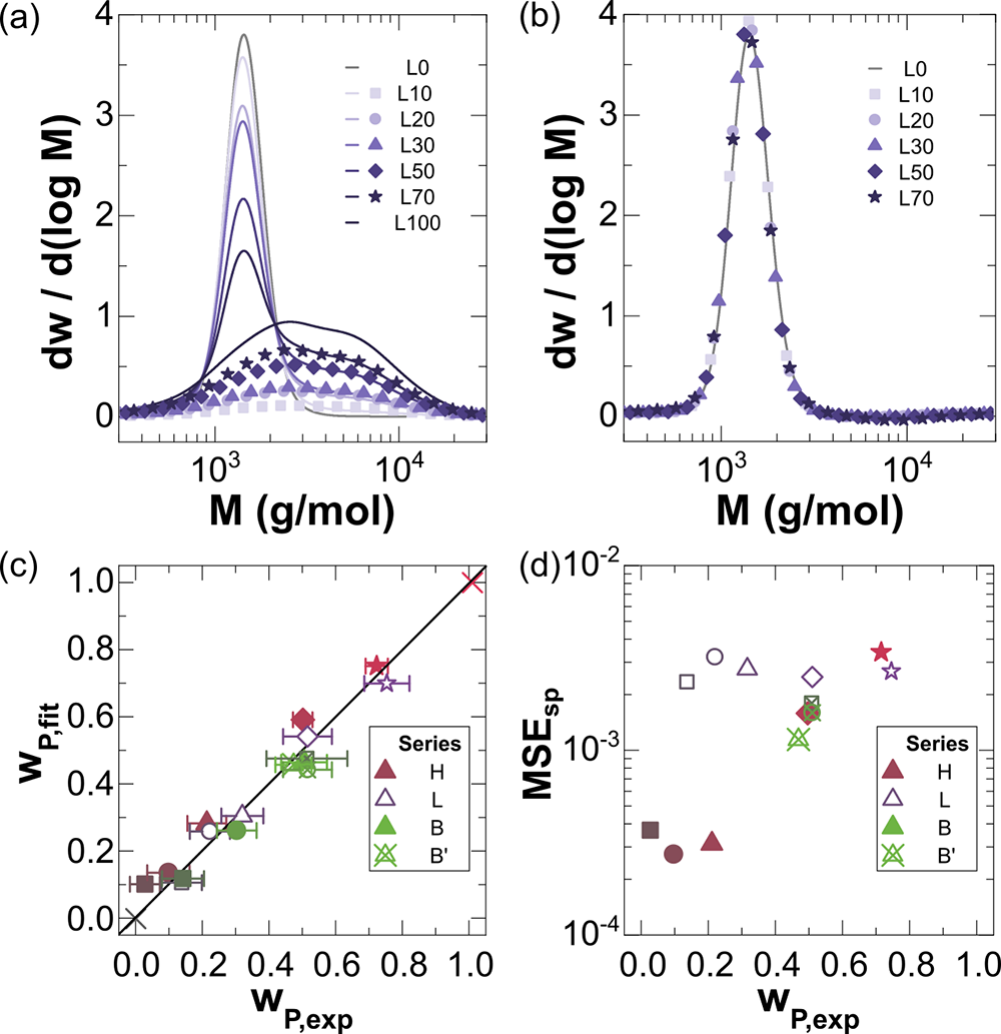
Molar mass (M) distribution analysis for surrogate blends. (a) Measured distributions (solid lines) in the L series with fits of the higher-molar-mass component for each blend (scatter points). (b) Resulting solid product distributions (scatter points) after subtraction of the higher-molar-mass component fit from (a) in comparison to the measured L0 distribution (gray line). (c) Parity plot of the fit intact weight fraction, *w*_p,fit_, versus the experimentally measured value, *w*_p,exp_. (d) Mean-squared errors (MSEs) between the solid product distributions and the known distributions to which they correspond for all measured blend series.

**Figure 3:**
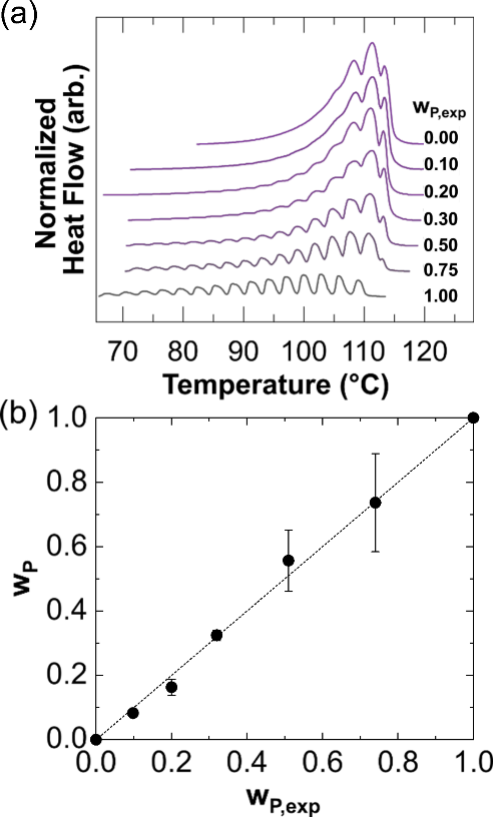
(a) Melting curves of thermally fractionated blends of LDPE_low_ and PW1000 (analogous to the L series) with the experimentally measured weight fraction, *w*_P,exp_, indicated for the LDPE component. (b) Parity plot of DSC-calculated (*w*_P_) and experimentally measured weight fractions (*w*_P,exp_).

To validate the GPC methodology, both low-molar-mass LDPE (LDPE_low_) and high-density polyethylene (HDPE) were used to simulate a starting feedstock polymer, and these PEs were blended with analytical standard PEs (PW500 and PW1000) that had lower molar masses and represented model solid deconstruction products. The weight-average molar mass (*M*_w_) and dispersity (*Đ*) of each PE shown in Figure 2 can be found in Table S1, and details of series names and blend compositions are listed in Table S2. As PE deconstruction progresses, the chains are expected to increase in branching degree; therefore, to validate the DSC methodology, PW1000 and LDPE_low_ represented a neat polymer feedstock and reacted polymer, respectively. PW1000 also served as a feedstock polymer for the DSC method, in contrast to its role as a deconstruction product for the GPC method due to its highly linear structure in comparison to LDPE_low_. The validations of the GPC and DSC methodologies were treated independently, allowing surrogate blends to include the same polymers used for different purposes. Blend compositions for the DSC methodology shown in Figure 3 are given in Table S3.

### Case Studies

To investigate the solids from LDPE hydrocracking, samples were obtained directly from Vance et al.^43^ The hydrocracking of LDPE occurred over a platinum tungstated zirconia catalyst at relatively low temperatures (250 °C) and 30 bar of H_2_, and the bifunctional catalyst was found to produce higher-value liquid alkanes. Additionally, the degree of isomerization of solid products was impacted by the relative amount of metal and Brønsted acid sites on the catalyst. The Pt nanoparticle diameter range was 1.1–1.8 nm,^43^ which does not explain the reactivity differences and changes to solid product distributions. Relevant catalyst compositions, reaction times, and solids conversions can be found in Supporting Information Section 2.13.

Pyrolysis of LDPE was carried out at 425 °C in an inert atmosphere via thermogravimetric analysis (TGA) (see the Supporting Information for experimental details), in which the gaseous products were discarded and unknown. The residual products after pyrolysis were assumed to be all solids, with no liquid fraction, because the melting temperatures were above 50 °C (Figure S17), which is expected, considering the high pyrolysis temperature that would either vaporize or gasify short chains. Rather than reaction time, the mass loss percentage was used to capture the kinetics of the reaction. Only samples up to 30% mass loss were analyzed, as the solids quickly degraded to low-molar-mass species.

## Results and Discussion

For this work, a combined GPC and DSC analysis methodology was developed to track the evolution of PE chains during deconstruction, accurately differentiating between the unreacted polymer and reaction products. Molar mass distributions from deconstruction often contain intact polymer, which is indicative of the efficacy of a given process (e.g., homogeneity of the reaction medium, external mass transfer limitations), and GPC is ideal for the analysis of such products. Instead of reducing a polymer’s molar mass distribution down to number-average and dispersity values, the entire distribution must be considered to gain insight into the relative weight fractions of chain sizes, as obtained via a series of component distributions and further partitioned into a solid product distribution and an intact distribution, both weighted by their fraction in the solid product stream (see Supporting Information, Sections 2.1–2.7). By blending PE samples with varying known molar mass distributions, this analysis can be validated. Each surrogate blend from the “L-series” exhibited signatures from PW1000 and LDPE_low_, as indicated by peaks at 1400 and 2600 g/mol, respectively. These molar mass distributions are shown in Figure 2a, wherein L100 refers to the distribution of neat LDPE_low_, and L0 is neat PW1000. As L100 (LDPE_low_) is the representative polymer feedstock for deconstruction, its distribution was known and was fit within all blend distributions (L10, L20, L30, L50, and L70). Example fits demonstrate that the high-molar-mass component (L100, Figure 2a) was effectively captured for all blend compositions. The molar mass distribution of solid deconstruction products (*F*_w,SP_), determined by the residual distribution after the high-molar-mass component was subtracted, agreed with the known L0 distribution (Figure 2b). This agreement for all blend compositions confirmed that the method successfully quantified the simulated, intact component within the experimental error of the weight fraction measurement (Figure 2c) and reproduced the low-molar-mass component (L0) within a low (<1%) mean-squared error (MSE) (Figure 2d). The successful demonstration of the GPC methodology for multiple sets of surrogate deconstruction blends established the potential quantification of intact polymer and solid deconstruction products in real hydroconversion mixtures.

Catalytic deconstruction reaction rates and product yields are informed by the relative mass fractions of the post-reaction gases, liquids, and solids, yet the solids also contain information about the reacted polymeric species present. Without the appropriate calibration standards, GPC cannot easily differentiate between various types of isomerized polymer chains, and even then, only provides an average branching frequency.^45^ On the other hand, DSC cannot directly identify molar mass changes in polymeric species; however, the branches along PE chains influence the melting behavior. The lamellar thickness and melting point of LDPE crystals are heavily dependent on the amount of copolymer (i.e., branch points) in the material.^46^ Because these branch points are excluded from the PE crystal,^47^ the distance between branch points on LDPE dictates the maximum lamellar thickness. Thus, an approach that leverages GPC to calculate the intact fraction and DSC thermal fractionation to calculate the reacted fraction is used to fully capture the key features of the solids.

Thermal fractionation via self-seeding and annealing (SSA) of PE crystals uses the crystal exclusion assumption to identify the distribution of branch points.^48,49^ SSA via DSC has shown good agreement with other more precise techniques for measuring branching architecture, such as temperature rising elution fractionation, but SSA requires significantly less time and no specialized equipment.^48,50,51^ Herein, the branching distribution of PEs was described by methylene sequence lengths (MSLs), which were estimated by the extrapolation of the known melting points for linear alkanes, and the normalized amount of a given MSL present in the material (i.e., relative heat of fusion).^52,53^ Thus, for a thermally fractionated polymer blend, the branching architectures of the neat components, normalized by their weight fractions, combine to form the architecture of the blend. To test this approach, branching distributions of blended LDPE_low_ and PW1000 (Table S3) were used to fit relative weight fractions of each component, and Figure 3a shows the melting curves after the SSA protocol (see the Supporting Information, Section 2.8–2.10) was applied to each sample. Neat LDPE_low_ (*w*_P,1.00_) had a broad range of melting peaks from 70 to 110 °C, whereas neat PW1000 (*w*_P,0.00_) had three strong melting peaks at relatively higher temperatures, which suggested the PW1000 is more linear (i.e., contains fewer branch points) than LDPE_low_. There was good agreement between the experimental weight fractions of the blends and the DSC-calculated weight fractions, demonstrating the applicability of SSA for blend composition analysis on the basis of branching architecture differences (Figure 3b). Given this successful surrogate blend approach, obtaining branching distributions via DSC can facilitate the deconvolution of multiple polyolefin species within a sample and therefore identify the reacted polymer content.^54^

In real hydroconversion solids, changes in branching and molar mass are expected to coexist such that there is a need to combine the GPC and DSC approaches to determine the composition of chains that have the same branching structure as the feedstock polymer, which is not always equal to the fraction of chains with the same molar mass distribution as the feedstock polymer. By comparing the insights gained from this methodology, we can elucidate how the polymer structure influences mechanistic deconstruction pathways and product species (Figure 1). Two prevalent valorization strategies of LDPE, namely, hydrocracking and pyrolysis, showcase the efficacy of employing the described GPC and DSC approach. Hydrocracking involves heterogeneous catalysis, which requires mass transfer to the catalyst surface to initiate the reaction. Additionally, hydrocracking enables multiple pathways by which chains can be isomerized, broken, or both.^43^ Conversely, pyrolysis is a noncatalytic, bulk, thermal reaction that typically involves the formation of radicals^55^ and can lead to a complex branching structure in the solids (i.e., the species left behind in the open reactor).^56^ Thus, the main difference between hydrocracking and pyrolysis, in the context of polymeric solid transformations, is the locality in which the reaction occurs (i.e., on the catalyst surface versus in the bulk), which is shown herein to greatly influence the product.

**Figure 4:**
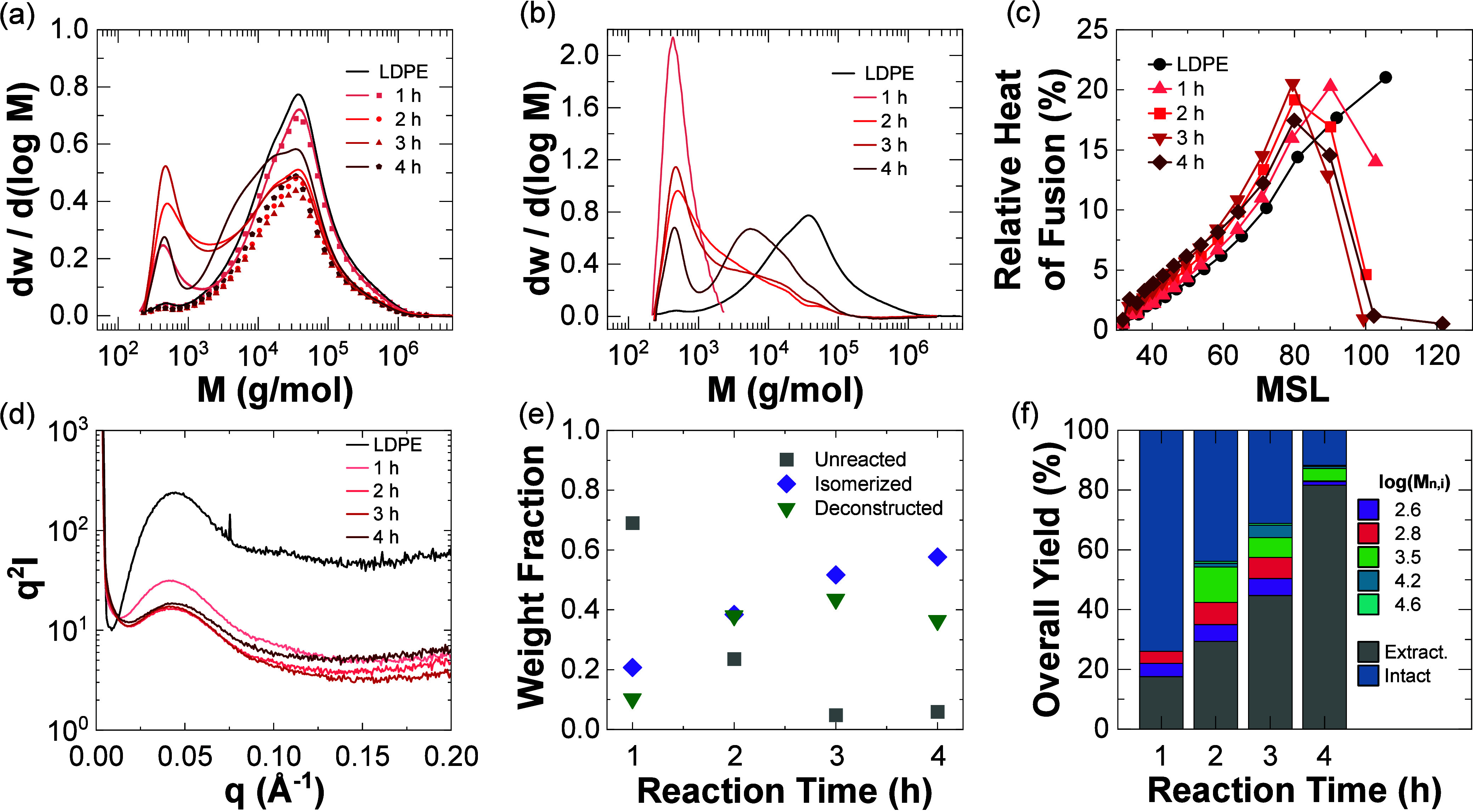
Evolution of LDPE hydrocracking solids over time. (a) Measured molar mass distributions (lines) and fits of the intact fraction (symbols) and (b) calculated solid product distributions. (c) Relative heats of fusion as a function of MSL. (d) Lorentz-corrected SAXS curves. Solids composition analysis in terms of chain types (e) and yields of distinct chain-length populations in the entire reaction mixture (f).

The characterization of the chain populations or branching architecture performed on the LDPE hydrocracking solids obtained from Vance et al.^43^ is shown in Figure 4. As expected, an overall decrease in the intact fraction (*w*_P_) occurred with an increasing reaction time (Figure 4a). Qualitatively, this behavior was evidenced by a decrease in intensity of the peak molar mass at ∼40000 g/mol. Additionally, there was an increase in the fraction of intermediate chain lengths (∼5000 g/mol) in the solid deconstruction products over reaction time (Figure 4b). Very few intermediate chain lengths were present after 1 h, suggesting that smaller chains may react first with the catalyst or larger chains may react several times. Thus, the average reacting polymer chain decreased in molar mass significantly before exiting the influence of the catalyst, by either desorption or multiple readsorption and reaction events. The observed rise in intermediate chain lengths and decline in smaller chain lengths (<1000 g/mol) indicates that smaller chains initially present in the reaction may have diminished sufficiently in size to become part of the extractables stream (<500 g/mol) and are no longer present in the solids.

The branching structure of neat LDPE was most significantly characterized by the largest MSL of 105, and as reaction time increased, the fraction of chains of this sequence length decreased but did not disappear (Figure 4c). At times longer than 2 h, the second longest MSL (90) decreased as well. These more-linear portions of the LDPE backbone may be more likely to interact with the catalyst surface, reacting more than small MSL portions (i.e., densely branched segments); this trend is analogous to that found in the catalytic hydrogen–deuterium exchange of branched polyolefins.^57−59^ Thus, the unreacted LDPE accounted for all MSL = 105 present in the solids, as both isomerization and chain scission will reduce MSL, as occurred during the course of the reaction. Although additional branching in PE samples may lower the melting point of similar MSLs, the extent of this decrease was not expected to be substantial.^46^ The MSLs reported may not be highly precise, as they are extrapolated from linear alkanes,^52^ and the branch length also affects melting point,^60^ but this dependence is not expected to be strong and other artifacts in the DSC, such as thermal lag^61^ or crystal reorganization,^62^ will complicate the analysis. To rectify this melting-point dependence, if the longest MSL of the solids was still higher than the second longest MSL of the pristine feedstock (90), then the calculated MSL was assumed to be unreacted chain segments, which limited reliance on exact MSLs for analysis (i.e., the relative fraction of the longest MSL is more important than the length). The branching distribution cannot be calculated for individual chains, and longer chains may possess multiple sequences of 105 non-branched carbons; therefore, the unreacted fraction calculated from DSC indicated the maximum value (actual unreacted fractions may be lower) because it was a segment-level, rather than a chain-level, representation. To verify the changes in branching architecture and extrapolated MSLs, small-angle X-ray scattering (SAXS) was performed (Figure 4d). A small but consistent shift to higher peak scattering vectors (*q*) suggested an overall smaller distribution of crystals. Lorentz-corrected intensities (*q*^2^*I*)^63^ in SAXS indicate crystal sizes of 50 Å for neat LDPE, similar to other SSA-fractionated LDPEs.^64^ Hydrocracking LDPE led to a decrease in crystal size that plateaued at 35 Å after 2 h of hydrocracking. Assuming a chain-tilt of 35°,^65,66^ the average lamellar thicknesses were 65 and 43 Å, for neat LDPE and 4 h hydrocracked solids, respectively. The average lamellar thicknesses via SAXS agreed well with the distributions of MSLs, which decreased after hydrocracking. Crystal size decreases will influence several mechanical properties of PEs, thus affecting the applications of the solids,^67^ which was revealed through thermal fractionation along with insight into chain linearity and polymer–catalyst interactions.

With the intact LDPE and unreacted LDPE calculated via GPC and DSC, respectively, the relative amounts of unreacted, isomerized, and deconstructed LDPE can be calculated via eq S3. As expected, the fraction of unreacted LDPE decreased with reaction time, and the fractions of isomerized and deconstructed LDPE both increased (Figure 4e). These fractions are more descriptive of the overall reaction progress rather than a weight fraction of total solids, which can imply unreacted chains. The viscosity of LDPE at 250 °C (127 Pa s) was 4 orders of magnitude higher than those of LDPE_low_ (2 × 10^–2^ Pa s) and liquid alkanes at 25 °C (∼10^–3^ Pa s;^68^ viscosity expected to be even lower at 250 °C). Diffusion coefficients of polymer chains are inversely proportional to viscosity;^69^ therefore, chain-level diffusion was expected to be a prominent contributing factor to the presence of unreacted chains after 4 h and a total solids conversion of 83% (Table S4). Additionally, the entire distribution of deconstruction solids was fit via a series of log-normal distributions (Figure 4f, Figure S10). As emphasized earlier, this approach allowed for more detailed quantification than solely using the average molar masses (i.e., moments of the entire distribution). These component distributions highlighted the increase in intermediate chain lengths at 2 and 3 h reaction times, with shorter chain lengths disappearing at longer reaction times (4 h). The overall composition of the reaction mixture broadened with increasing reaction time (i.e., more log-normal distributions were required to describe the molar mass distribution). This chain population heterogeneity was attributed to an increase in the number of molecules able to undergo chain scission as more species became mobile. However, after 4 h, there was a decrease in product diversity as these species became small enough to enter the extractable stream. Thus, the combined approach utilizing GPC and DSC facilitated a comprehensive understanding of the LDPE deconstruction process, revealing dynamic shifts in unreacted, isomerized, and deconstructed LDPE fractions over time. These findings suggest that catalysts should be chosen on the basis of polymer branching, with linear-interacting catalysts at low solids conversions being exchanged for branch-interacting catalysts at high solids conversions. Reaction conditions (e.g., temperature, time) should be adjusted to account for polymer diffusion limitations and varying feedstock viscosities.

**Figure 5:**
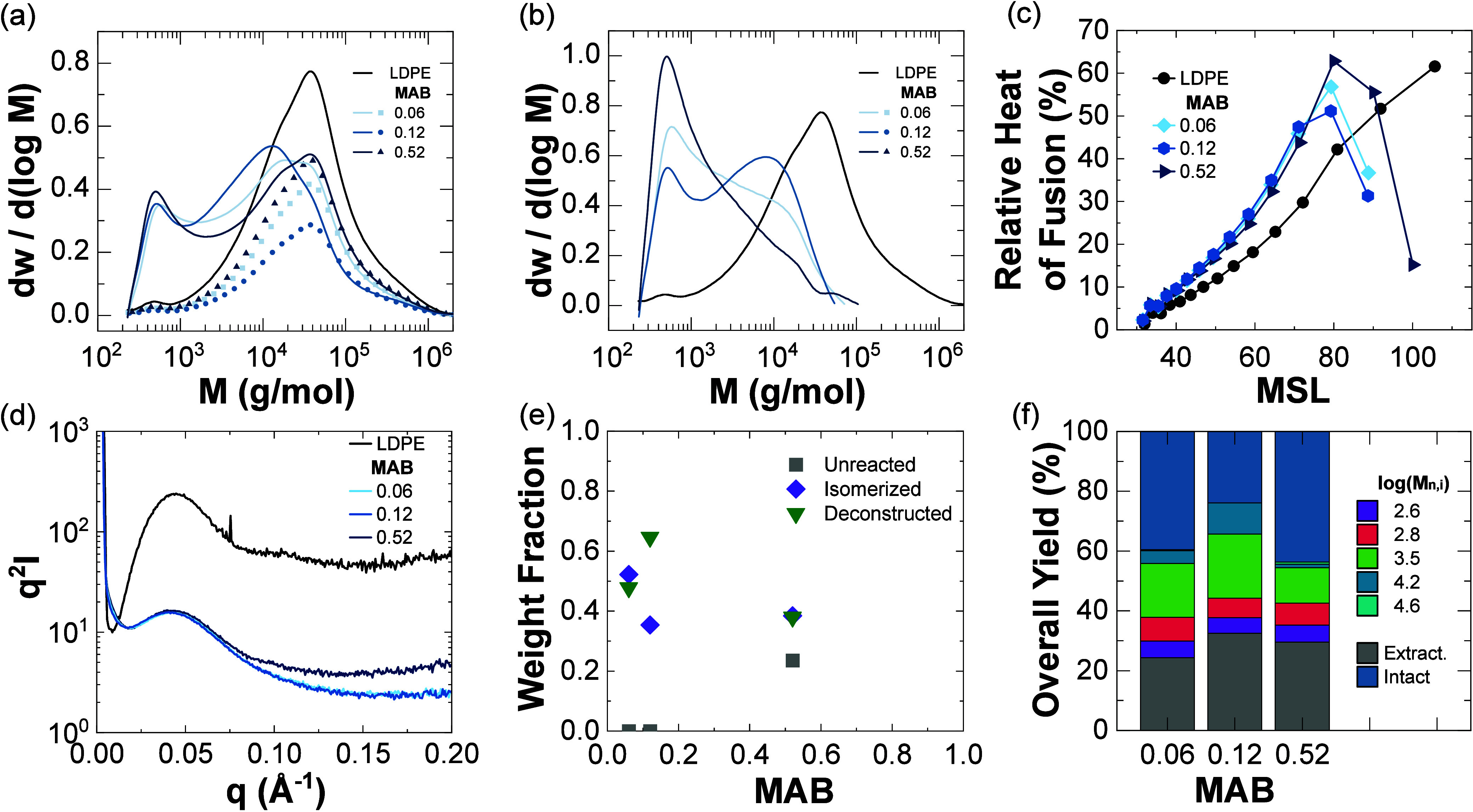
Solids characterization results for the effect of catalyst MAB on the hydrocracking of LDPE. (a) Measured molar mass distributions (lines) and fits of the intact fraction (symbols) and (b) calculated solid product distributions. (c) Relative heats of fusion as a function of MSL. (d) Lorentz-corrected SAXS curves. Solids composition analysis in terms of chain type (e) and yields of distinct chain-length populations in the entire reaction mixture (f).

In addition to the changes over reaction time, the hydrocracking catalyst MAB has been associated with alterations in the polymer branching content of the solids but exhibited a limited impact on branching within the liquids.^43^ To adjust for MAB effects, independent of reaction time, the LDPE was hydrocracked for 2 h at different MABs (Figure 5). Unlike the relationship between unreacted LDPE and reaction time, there was a non-monotonic dependence of the LDPE fractions with MAB (Figure 5a). Notably, at a high MAB (0.52), solid deconstruction products were composed of more low-molar-mass species (<1000 g/mol) (Figure 5b), and a high MAB also led to more intact LDPE. As described above, a highly isomerized LDPE has increased branching points, which decreased polymer activity with the catalyst, thus leading to a broadened molar mass distribution. However, the branching architectures and SAXS of the solids did not significantly depend on the MAB (Figure 5c). Additionally, particularly little unreacted LDPE was retained for low MAB, and similar deconstructed and isomerized LDPE fractions existed as a function of MAB (Figure 5e). As MAB increased, isomerization events should have increased, but the isomerized LDPE fraction remained between ∼0.35 and ∼0.55. The species in the reactor may have phase separated, and depending on when phase separation occurred, interactions with the catalyst could have changed.^34^ The MAB did not significantly affect either the solids conversion or fraction of branched isomers in the extractables stream, but increasing MAB led to increased C_21+_ wax-range products.^43^ Competitive adsorption between small alkanes with polymer chains to catalyst sites was hypothesized to contribute to the increased wax-range products, and the GPC and DSC results suggested that the competitive adsorption can be further separated into highly linear and highly branched polymer chains. The increased isomerization reactions led to chains more likely to desorb from the catalyst prior to near-complete chain scission and created more intermediate molar mass species. Moreover, with no clear trend in the log-normal distributions of product species (Figure 5f), the influence of small or intermediate chain lengths was difficult to discern. The described methodology cannot directly detect the interplay between the miscibility of the medium, reaction mechanism, and other physical phenomena (e.g., adsorption, diffusion) within this MAB series.

**Figure 6:**
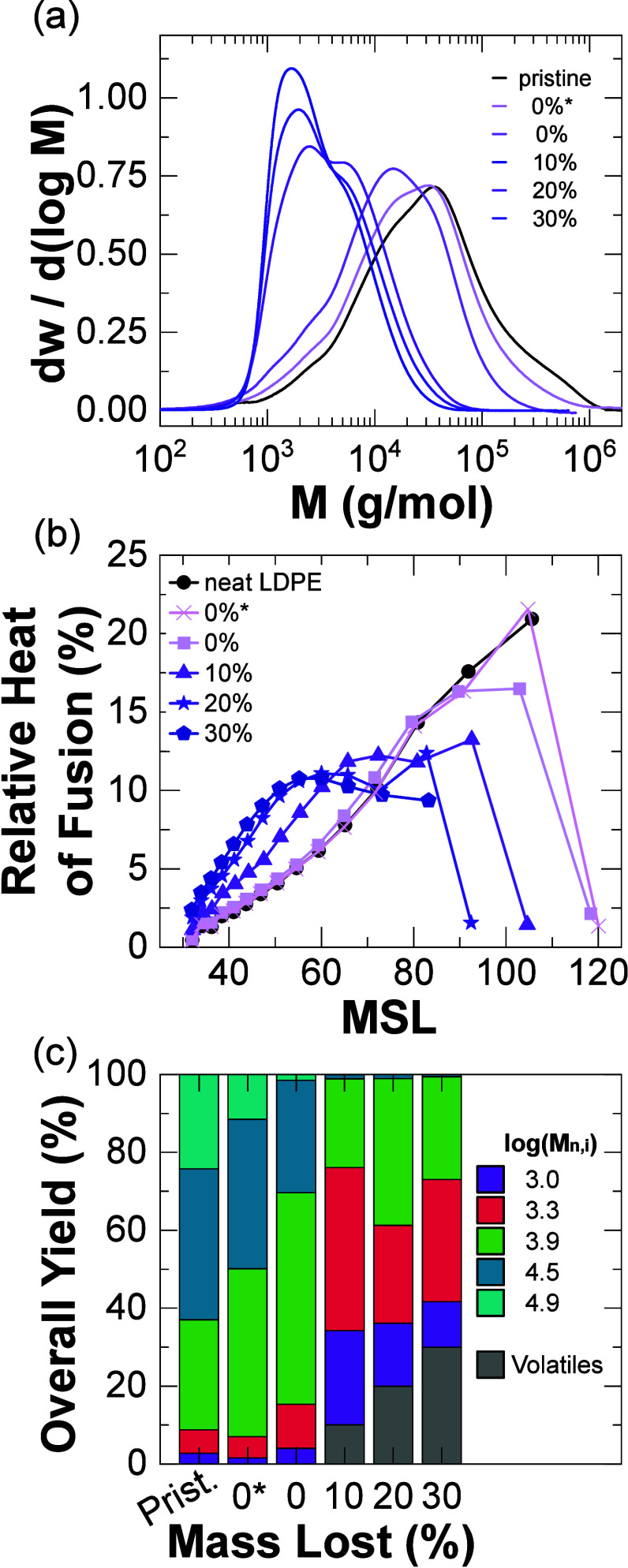
Solids characterization results for the pyrolysis of LDPE at different mass losses. (a) Measured molar mass distributions. (b) Relative heats of fusion as a function of MSL. (c) Overall (mass) yields of distinct chain-length populations. All data are from experiments at 425 °C except for the 0%* data, for which the experiment was stopped at 400 °C during the temperature ramp phase.

As previously mentioned, another PE valorization strategy is pyrolysis. In comparison to hydrocracking, pyrolysis occurs mostly homogeneously in the bulk polymer melt (i.e., mass diffusion is not required to react) rather than heterogeneously. Any diffusion of vaporizable products and heat transfer limitations associated with pyrolysis^70−72^ were assumed to be negligible factors in the deconstruction of polymer chains, given the relatively small sample sizes (<20 mg) and pyrolysis reaction times (10^2^–10^3^ s). Solids from all pyrolyzed samples revealed a complete absence of intact and unreacted LDPE. The assumptions (Supporting Information, Section 2.4) of the GPC fits were violated, in which chain reactivities appeared to depend on molar mass; consequently, the degradation process immediately altered the distribution of the feedstock without affecting the total mass. Approximately a 1/2 order of magnitude decrease in molar mass occurred before the reaction temperature (425 °C) was reached (Figure 6a). To highlight the fast molar mass decrease, LDPE was pyrolyzed at 400 °C for 0 s (i.e., brought to the reaction temperature and immediately cooled). Even though the mass loss was less than 0.5%, the sample displayed a significant drop in molar mass, particularly in the high-molar-mass tail of the distribution (>100000 g/mol). The peak molar mass decrease and the limited product diversification (Figure 6c) was consistent with random scission events, predicted via population balance models.^73,74^

Interestingly, branching architecture evolved similarly to hydrocracking in which the largest MSL fraction decreased with time (i.e., increased mass loss); however, a more condensed MSL distribution was produced (Figure 6b). As there was no intact LDPE fraction measured via GPC, and the intact LDPE comprised unreacted and isomerized LDPE chains, no unreacted LDPE existed within the pyrolysis solids. The reaction mixture composition of log-normal distributions showed a sharp decrease in the fraction of high-molar-mass species and shifted to a larger fraction of distributions centered at ∼1000 and ∼2000 g/mol (log *M* = 3.0 and 3.3, respectively) (Figure 6c). The consistent shift in molar mass species and branching architecture confirmed the bulk and semi-random nature of pyrolysis. Although solid products from LDPE pyrolysis exhibited thermal behavior similar to that of hydrocracked LDPE, the utilization of GPC underscores the distinctiveness of pyrolysis in comparison to hydrocracking, emphasizing the crucial role of elucidating differences in chain sizes present.

## Conclusions

To attain a comprehensive understanding of polymer populations within PE deconstruction reaction mixtures, especially solids, we implemented a combined GPC and DSC polymer analysis approach. Unlike previous methods that often require specialized instrumentation (e.g., solid-state nuclear magnetic resonance spectroscopy^43^) or lack detailed characterization of resulting polymeric species, our approach provided a more thorough accounting of the various polymer populations, including chain lengths and branching architectures. PE blends with predetermined molar masses and branching architectures were selected to validate the assumptions made in determining the intact and reacted polymer fractions using GPC and DSC, respectively. Although each technique individually offered valuable insights into the polymer species present in deconstructed solids, the combination allowed for enhanced product identification, distinguishing between unreacted, isomerized, and deconstructed fractions within the species that solidify at room temperature.

To highlight the utility of the approach, hydrocracked LDPE solids were obtained after various reaction times and catalyst compositions, and the solids exhibited distinct chain length and architectural transformations.^43^ Herein, we found that the most linear segments (i.e., the largest methylene sequence lengths of LDPE) were more likely to react with a hydrocracking catalyst, whereas the densely branched portions exhibited limited changes. Additionally, the relative concentration of low molar mass species (<1000 g/mol) increased before significant amounts of intermediate molar mass species were noted in the solids, which suggested almost complete chain deconstruction prior to diffusion away from the catalyst. In LDPE hydrocracking, the fraction of unreacted LDPE decreased with increasing reaction time, yet it was nonzero after 4 h. High MAB led to more intermediate-molar-mass species present in the solids, which indicated a competitive adsorption effect between highly linear and highly branched chains, but the effects of polymer adsorption kinetics, chain diffusion, and phase homogeneity, especially in the presence of highly branched LDPE, were difficult to discern. In comparison to hydrocracking, the bulk reaction of LDPE pyrolysis generated product species with gradually decreasing chain populations, and condensed branching structures, due to the absence of a polymer-chain-diffusion limitation and, notably, led to solids with no identifiable unreacted LDPE. The contrast observed in the solids from hydrocracking vs pyrolysis parallels that observed in the liquid products from these deconstruction methods, emphasizing the importance of proper quantification of deconstruction solids. This methodology also offers valuable insight into intrinsic polymer properties affecting catalytic deconstruction. By determining the evolution of polymer species, the PE deconstruction process can be understood and optimized for the changing reaction viscosities, phase behavior, and polymer–catalyst interactions, thereby improving reaction times and yields. Moreover, the refined identification of product species through TEA/LCA calculations can further advance sustainability efforts in plastics waste management.

## Supporting Information Available

Additional experimental details, materials, model assumptions, and methods and compiled GPC and DSC results (PDF)
