# Supplementary material for: Tracking Chain Populations and Branching Structure during Polyethylene Deconstruction Processes
Source: ACS Cent Sci. 2024 Aug 21;10(9):1755–64. doi: 10.1021/acscentsci.4c00951 (PMC11428289; doi:10.1021/acscentsci.4c00951)
Supplement: Supplementary file 1 — oc4c00951_si_001.pdf [file oc4c00951_si_001.pdf]

Supporting Information for:

Tracking Chain Populations and Branching Structure during Polyethylene Deconstruction Processes

Author List: Alex H. Balzer[a,b,†], Zachary R. Hinton[a,†], Brandon C. Vance[a,b], Dionisios G. Vlachos[a,b], LaShanda T.J. Korley[a,b,c,d,*], Thomas H. Epps, III[a,b,c,d,*]

[a]Center for Plastics Innovation (CPI), University of Delaware, Newark, Delaware, USA

[b]Department of Chemical and Biomolecular Engineering, University of Delaware, Newark, Delaware, USA

[c]Department of Materials Science and Engineering, University of Delaware, Newark, Delaware, USA

[d]Center for Research in Soft matter and Polymers (CRiSP), University of Delaware, Newark, Delaware, USA

[†]These authors contributed equally to this work.

*Corresponding authors

Email: lkorley@udel.edu, thepps@udel.edu

1. Experimental Section

1.1 Materials

Low-density polyethylene (LDPE, $LDPE_{low}$), high-density polyethylene (HDPE), and analytical standard polyethylenes (PW500, PW1000) were obtained from Sigma-Aldrich. Butylated hydroxytoluene (BHT, >99%, also known as 2,6-di-tert-butyl-4-methylphenol) was purchased from Sigma Aldrich. 1,2,4-trichlorobenzene (TCB, HPLC grade) was obtained from Fisher Scientific. The weight-average molar mass M_w, dispersity ($Đ$), and peak molar mass (M_{pk}) for all polymers are listed in **Table S1**. All solids from hydrocracking were obtained directly from Vance *et al.*[1], and the separation of the polymer solids from the catalyst is described in [1].

Table S1. Relevant macromolecular characterization of the blend components used in this work.[a]

	M_w	$Đ$	M_{pk}
	g/mol	-	*g/mol*
**LDPE**	1.0×10^5	6.3	5.9×10^4
**$LDPE_{low}$**	4.3×10^3	4.0	2.6×10^3
**HDPE**	6.4×10^4	5.4	3.4×10^4
**PW500**	6.5×10^2	1.2	7.8×10^2
**PW1000**	1.5×10^3	1.1	1.4×10^3

[a]Molar mass distributions were obtained *via* high temperature - gel permeation chromatography described in Section 1.2.

1.2 High Temperature - Gel Permeation Chromatography

High temperature - gel permeation chromatography (HT-GPC) was performed with a high-temperature system (Tosoh HLC-8312GPC/HT with three TSKgel GMH_{HR}-H(20)HT columns in series) equipped with refractive index (RI) and viscometry detectors. Samples were dissolved at a concentration of 1 mg/mL in TCB (with 500 ppm BHT) and heated for at least 2 h at 140 °C prior to injection. 300 µL injections of sample solutions were eluted at 0.8 mL/min in TCB at 140 °C.

A series of nine narrow standards of polystyrene (PS) (Tosoh PStQuick C and D) were used to calibrate the system for 2.5 < log M < 6.5. The calibration was adjusted using the Mark-Houwink relationship to account for differences in intrinsic viscosity between PS and polyethylene (PE):

$$\log M_{PE} = \left(\frac{1}{1+\alpha_{PE}}\right)\log\frac{K_{PS}}{K_{PE}} + \left(\frac{1+\alpha_{PS}}{1+\alpha_{PE}}\right)\log M_{PS} \tag{S1}$$

wherein M_{PE} is the peak molar mass of a PE sample; M_{PS} is the calibrated molar mass of the PS standard given by the calibration curve, and α and K are the Mark-Houwink parameters for each polymer. Values of α and K were obtained for both polymers in TCB at 140 °C: for PE, K_{PE} = 39 µL/g and α_{PE} = 0.725, and for PS, K_{PS} = 19 µL/g and α_{PS} = 0.655.[2]

The weight differential distribution functions, *W (log M)*, were determined using the standard method.[2] Yields of individual molar mass solids (Y_j) were determined from the RI detector response:

$$Y_j = x_j Y_s = Y_s \frac{RI}{\int RI \, \mathrm{d}R_t} \tag{S2}$$

wherein x_j is the mass fraction of a given slice, Y_s is the total solids yield, RI is the detector response, R_t is the retention time, and the integration is over the entire sample peak.

1.3 Differential Scanning Calorimetry

Differential scanning calorimetry (DSC) was performed using a Discovery DSC instrument (TA Instruments). Approximately 5 mg of each sample was sealed in an aluminum crucible, and the temperature was controlled between 0 °C and 150 °C at a ramp rate of 10 or 20 °C/min under nitrogen flow. Temperatures chosen for the self-seeding and annealing (SSA) protocol are described in more detail in Section 2.8. All DSC plots are oriented as endotherm up.

1.4 Thermogravimetric Analysis

Pyrolysis of LDPE samples was performed using a TGA 5500 instrument (TA Instruments). Approximately 15 mg of each sample was placed on a platinum crucible and heated under nitrogen flow from 30 °C to the pyrolysis temperature at 50 °C/min. Once the pyrolysis temperature was reached, an isothermal hold was applied until the desired mass loss occurred, after which time the sample was cooled to 30 °C at 50 °C/min.

1.5 Small-angle X-ray Scattering

Small-angle X-ray scattering (SAXS) was conducted on a Xenocs instrument with a sealed-tube X-ray source (Cu Kα, λ = 1.54 Å) operating at 2.0 kW with a Dectris Pilatus 300k 2-D detector (3 panels, 48- chip array, 1 pixel = 172 x 172 μm^2). The path tubes were held under dynamic vacuum to reduce scattering from air, and the sample-to-detector distance was 1200 mm. All samples were measured with two exposures of 5 min each at different detector positions and stitched together using the instrument's line-eraser function to remove blind spots in the 2-D spectrum. All 2-D scattering data were azimuthally integrated, resulting in plots of Lorentz-corrected intensity (q^2I) vs. scattering vector (q).[3]

1.6 Oscillatory Rheology

The viscosity was quantified using a strain-controlled, torsional Ares G2 rheometer (TA Instruments). Polymer samples were loaded into the test geometry (25-mm-diameter parallel plates for LDPE, 50-mm-diameter parallel plates for $LDPE_{low}$) that had been preheated to 250 °C. The

samples were slowly pressed and trimmed, such that the final thickness was 1 mm (25 mm plates) or 0.5 mm (50 mm plates). A pre-shear step was applied at 1 rad s^{-1} and 0.2% strain for 60 s to relax any stresses arising from sample loading. The strain amplitude remained within the linear viscoelastic regime (as determined by amplitude sweeps) and was selected to be 5% for LDPE and 25% for $LDPE_{low}$. A frequency sweep (between 0.1 and 500 rad s^{-1}) was performed to obtain linear viscoelastic responses at 250 °C. All experiments were conducted in a nitrogen atmosphere.

2. Additional Results and Discussion

To investigate the validity of the combined HT-GPC and DSC approach, blends of PEs with varying molar masses and branching densities were used as surrogates for the solids from deconstruction. Low molar mass PEs were treated as deconstruction products from a higher molar mass PE feedstock (HT-GPC methodology), and branched PEs were treated as reacted (isomerized or deconstructed) products from linear PE feedstock (DSC methodology). For intact polymer weight fractions, the molar mass distribution for the high molar mass PE feedstock was scaled to fit within the surrogate deconstruction blend molar mass distribution, and for the unreacted polymer weight fraction, the heat of fusion for the linear PE feedstock was scaled to fit within the thermally-fractionated deconstruction blend melting trace. The governing mass balances of polymer species present and the assumptions to the fits are described herein.

2.1 Blend compositions for HT-GPC Methodology

The hydrocracking and pyrolysis experiments (**Figures 4-6**) used LDPE, whereas proof-of-concept experiments, which demonstrated the methodology, shown in **Figures 2 and 3** and

described in SI Sections 2.1-2.8, used $LDPE_{low}$. Experimentally measured weight fractions of the surrogate blends applied in the HT-GPC and DSC experiments are listed in **Table S2**.

Table S2. Composition (w/w) of the surrogate blends used in the HT-GPC experiments.

	**PW500**	**PW1000**	**$LDPE_{low}$**	**HDPE**
**H3**	-	-	0.97	0.03
**H10**	-	-	0.90	0.10
**H20**	-	-	0.79	0.21
**H50**	-	-	0.50	0.50
**H70**	-	-	0.28	0.72
**H100**	-	-	-	1.00
**L0**	-	1.00	-	-
**L10**	-	0.86	0.14	-
**L20**	-	0.78	0.22	-
**L30**	-	0.68	0.32	-
**L50**	-	0.49	0.51	-
**L75**	-	0.25	0.75	-
**L100**	-	-	1.00	-
**B0**	1.00	-	-	-
**B10**	0.42	0.44	0.14	-
**B30**	0.34	0.36	0.30	-
**B50**	0.27	0.23	0.50	-
**HB25**	0.26	0.27	0.21	0.26

2.2 Mathematical Basis for the HT-GPC Methodology

Given a typical closed reaction system that converts pristine feedstock into product streams that phase separate at analysis conditions, a material balance can be performed around the system boundaries (as depicted in **Figure S1**). Hydrocracking and other deconstruction processes often have been characterized by extractable and solid yields (Y_{ex} and Y_s, respectively) defined on a carbon basis, and that convention is followed herein.

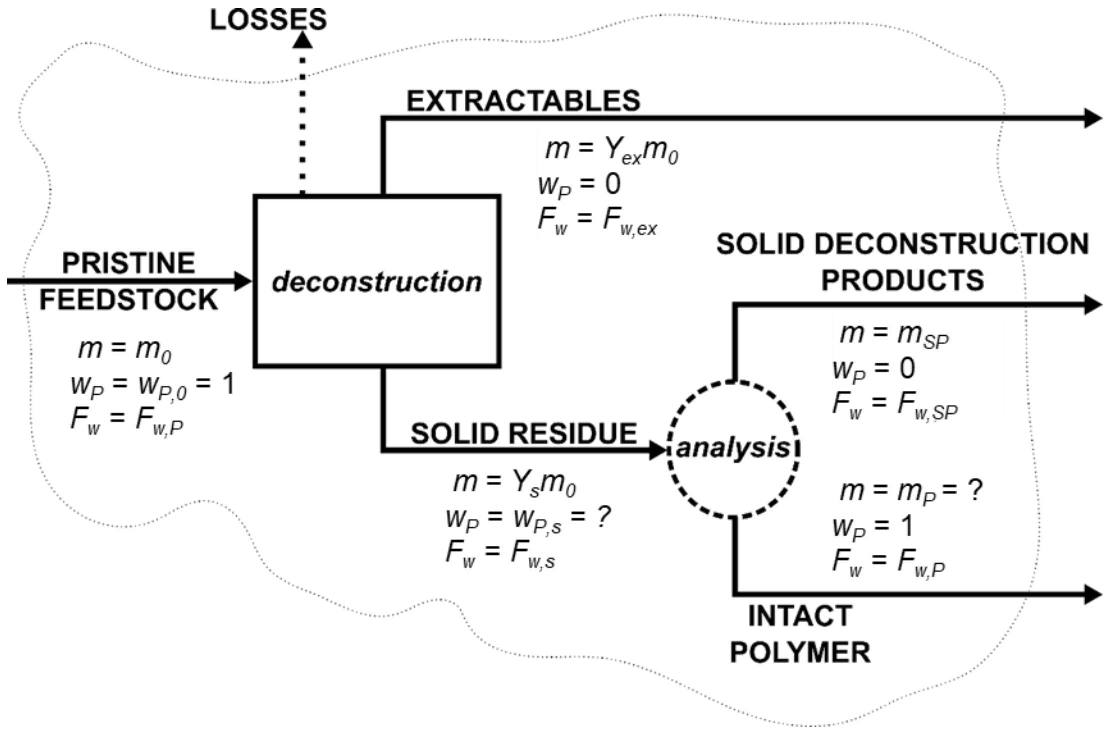

Figure S1. Schematic of the material balance performed around the process, wherein the inlet stream is converted to the outlets over a fixed period of time. Stream details are given by: m, mass; w_P, the weight fraction of intact (*i.e.*, pristine molar mass) polymer; and F_w, the differential weight fraction distribution (as a function of *log M*). The dotted line represents the control volume considered for the material balance.

The material balance for intact polymer can be written as:

$$m_0 = m_{ex} + m_{SP} + m_P \quad \textbf{(S3)}$$

wherein m_0 is the initial mass of pristine feedstock, m_{ex} is the mass of the liquid and gas products (*i.e.*, extractables), m_{SP} is the mass of the solid deconstructed products, and m_P is the mass of the intact polymer products. Performing the material balance around the analysis block, one can define the intact polymer mass fraction ($w_{P,s}$);

$$w_{P,s} = \frac{m_P}{m_s} = \frac{m_P}{Y_s \cdot m_0} \quad \textbf{(S4)}$$

however, both sides of the equation are unknown. A mole balance that considers the reaction of species (including a conversion) would traditionally be used to solve the system of balances, but

this approach is cumbersome for disperse chain populations with unknown conversions. In fact, it is this strategy that is desirable and motivates the development of our method. The additional balance can be derived from the product distributions. The generalized definition of the differential weight fraction distribution is:

$$\int_0^\infty F_w \, d(\log M) = \int_0^\infty \frac{dw}{d(\log M)} \, d(\log M) = 1 \tag{S5}$$

From this point forward, the limits of integration will be dropped and are assumed to be the entire range of M. Therefore, one can write the mass balance around the analysis block as:

$$m_s = m_{SP} + m_P \tag{S6}$$

and

$$m_s \int F_{w,s} \, d(\log M) = m_{SP} \int F_{w,SP} \, d(\log M) + m_P \int F_{w,P} \, d(\log M) \tag{S7}$$

Utilizing **(S4)**, the relationship can be rewritten as:

$$\int F_{w,s} \, d(\log M) = \left(1 - w_{P,s}\right) \int F_{w,SP} \, d(\log M) + w_{P,s} \int F_{w,P} \, d(\log M) \tag{S8}$$

To return the differential weight fraction distribution, a derivative is taken and written as:

$$\begin{aligned} \frac{d}{d(\log M)} \left\{ \int F_{w,s} \, d(\log M) \right\} \\ = \left(1 - w_{P,s}\right) \frac{d}{d(\log M)} \left\{ \int F_{w,SP} \, d(\log M) \right\} \\ + w_{P,s} \frac{d}{d(\log M)} \left\{ \int F_{w,P} \, d(\log M) \right\} \end{aligned} \tag{S9}$$

According to the fundamental theorem of calculus, this equation can be rewritten as:

$$F_{w,s} = \left(1 - w_{P,s}\right)F_{w,SP} + w_{P,s}F_{w,P} \quad \textbf{(S10)}$$

The measured solids distribution can be decomposed into a solid product distribution and an intact distribution, both weighted by their weight fraction in the solid product stream. In this manuscript, $w_{P,s}$ will be referred to as w_P, as it is the only value of w_P that is neither unity nor zero. Because the left-hand side of **(S10)** is a measured quantity, and $F_{w,P}$ is known, the only unknowns are the values of interest: $F_{w,SP}$ and w_P.

Alternatively, it may be desirable to operate on a function with constrained values, such as the cumulative weight fraction function, W_{cum}, which can be defined as:

$$W_{cum}(\log M_i) = \int_0^{M_i} F_w \, d(\log M) \quad \textbf{(S11)}$$

$$\frac{d}{d(\log M)}\left[W_{cum}\right] = \frac{dw}{d(\log M)} = F_w \quad \textbf{(S12)}$$

Thus, an alternative to **(S9)** can be written as:

$$\begin{aligned} &\int \left\{\frac{d}{d(\log M)}\left[W_{cum,s}\right]\right\} d(\log M) \\ &= (1 - w_P) \int \left\{\frac{d}{d(\log M)}\left[W_{cum,SP}\right]\right\} d(\log M) \\ &+ w_P \int \left\{\frac{d}{d(\log M)}\left[W_{cum,P}\right]\right\} d(\log M) \end{aligned} \quad \textbf{(S13)}$$

Again, the fundamental theorem of calculus leads to a form rewritten as:

$$W_{cum,s} = (1 - w_P)W_{cum,SP} + w_P W_{cum,P} \quad \textbf{(S14)}$$

Assuming that no polymer chains longer than those in the original feedstock are generated, there is a value of molar mass (M_{fit}) that is the threshold beyond which there are no polymer chains that are solid deconstruction products, and that value can be written as:

$$\lim_{M \to M_{fit}} F_{w,SP} = 0 \quad \textbf{(S15)}$$

$$\lim_{M \to M_{fit}} W_{cum,SP} = 1 \quad \textbf{(S16)}$$

and, therefore, **(S10)** and **(S14)** can be interpreted as:

$$\lim_{M \to M_{fit}} F_{w,s} \approx w_P F_{w,P} \quad \textbf{(S17)}$$

$$\lim_{M \to M_{fit}} W_{cum,s} \approx (1 - w_P) + \ w_P W_{cum,P} \quad \textbf{(S18)}$$

These formulations are central to this work. Given this assumption, **(S10)** is tractable, and the material balance can be fully determined. Additionally, it was assumed that there was no molar mass-dependent polymer chain reactivities. Qualitative analysis of the entire molar mass-distribution confirmed there were no signatures of such dependence (*i.e.*, no disappearance of certain molar mass fractions prior to others). This observation – along with reported melt-adsorption behavior of polymers, wherein the chains adsorb in formations of trains, loops, and tails, but the entire chain is never fully adsorbed[4-10] – challenges the possibility of a molar mass-dependent reactivity. Chain-length reactivities were treated as separate from mass diffusion limitations and competitive adsorption, in which a polymer of lower molar mass will diffuse to the reactive surface faster, and larger chains adsorb more strongly to surfaces,[5, 11] yet once the chain reaches the catalyst, the reaction kinetics will be constant. The reaction and monomer adsorption/desorption timescales also were assumed to be much faster than chain diffusion.[10, 12]

To determine the value of w_P, **(S17)** or **(S18)** still needs to be solved. Because $F_{w,P}$ and $F_{w,s}$ are many-valued functions that are fixed, **(S17)** and **(S18)** require a minimization (fit) to solve, defined as:

$$\min_{w_P} \left| F_{w,s} - w_P \, F_{w,P} \right|_{M > M_{fit}} \quad \textbf{(S19)}$$

$$\min_{w_P} \left| W_{cum,s} - (1 - w_P) - \; w_P F_{w,P} \right|_{M > M_{fit}} \quad \textbf{(S20)}$$

Once an optimal value of w_P is determined, the solid deconstruction product distribution can be defined as:

$$F_{w,SP} = \frac{F_{w,s} - w_P F_{w,P}}{1 - w_P} \quad \textbf{(S21)}$$

$$W_{cum,SP} = \frac{W_{cum,s} - w_P W_{cum,P}}{1 - w_P} \quad \textbf{(S22)}$$

and the reconstructed differential weight fraction distribution can be written as:

$$F_{w,SP} = \frac{d}{d(\log M)} \left[W_{cum,SP} \right] \quad \textbf{(S23)}$$

In the fit routine, a value for M_{fit} is not known *a priori*. It is expected that too small a value would lead to significant interference from solid product species, such that **(S15)** does not hold, and too large of a value will affect convergence to a meaningful solution. Therefore, we tested the effect of M_{fit} on the estimation of w_P using two surrogate blends, H3 and H50 (**Table S2**). As shown in **Figure S2**, it is possible for both over- and under-shooting of the portion of the measured distribution corresponding to the intact polymer. The mean-squared error of these fits (**Figure S2c**) demonstrates a minimum exists at $M_{fit} = M_{pk}$. The values of w_P obtained from these fits (**Figure S2d**) show that values of M_{fit} in close proximity (within one order of magnitude) to M_{pk} lead to errors (from the optimal value) similar to the experimental error in w_P. Therefore, we have

concluded that M_{fit} should be set equal to M_{pk}, which is convenient considering that, in real uses of this approach, no clear indication of M_{fit} would be given, but M_{pk} of the feedstock could be measured.

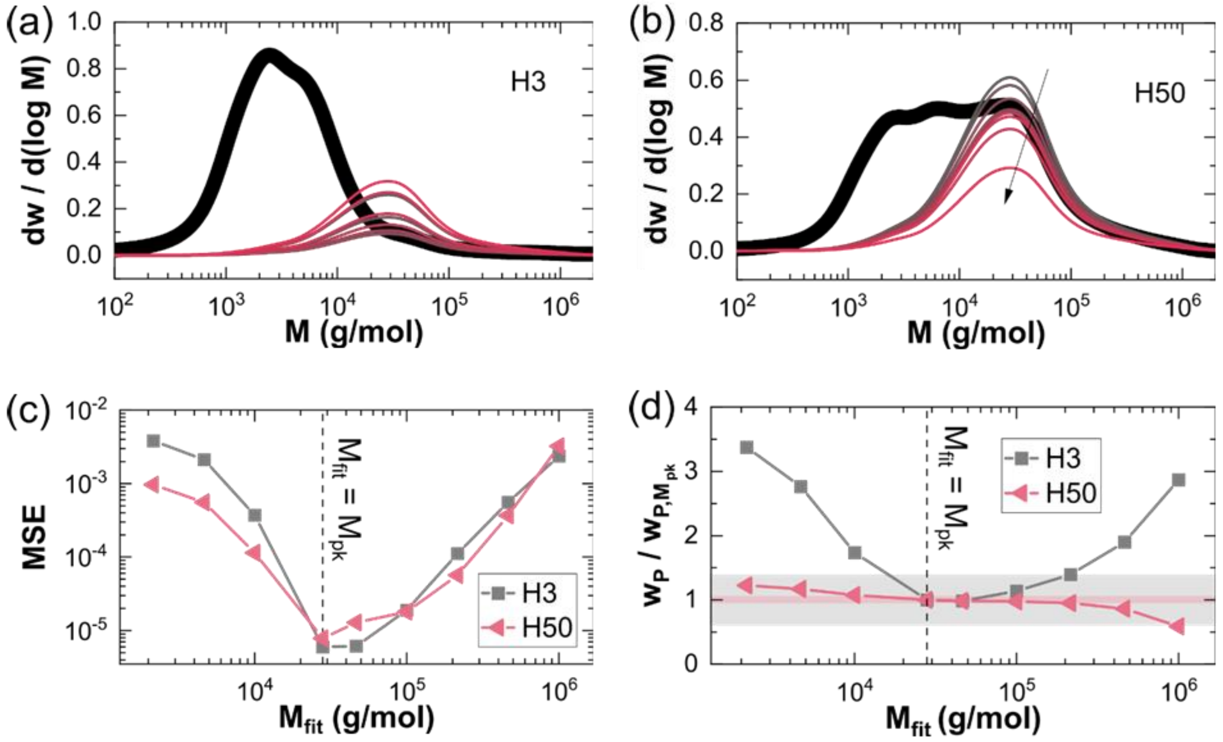

Figure S2. Example behaviors for varying M_{fit}. (a, b) Measured distributions (points) with fits utilizing increasing M_{fit} values indicated by color (from grey to pink). (c) Mean squared error (MSE) of the fit and (d) fractional w_P (normalized by the value at M_{pk}) as a function of M_{fit}. Shaded regions in (d) represent one normalized standard deviation from the experimental measurement of mass fraction placed around unity.

2.3 Demonstration of the HT-GPC Methodology

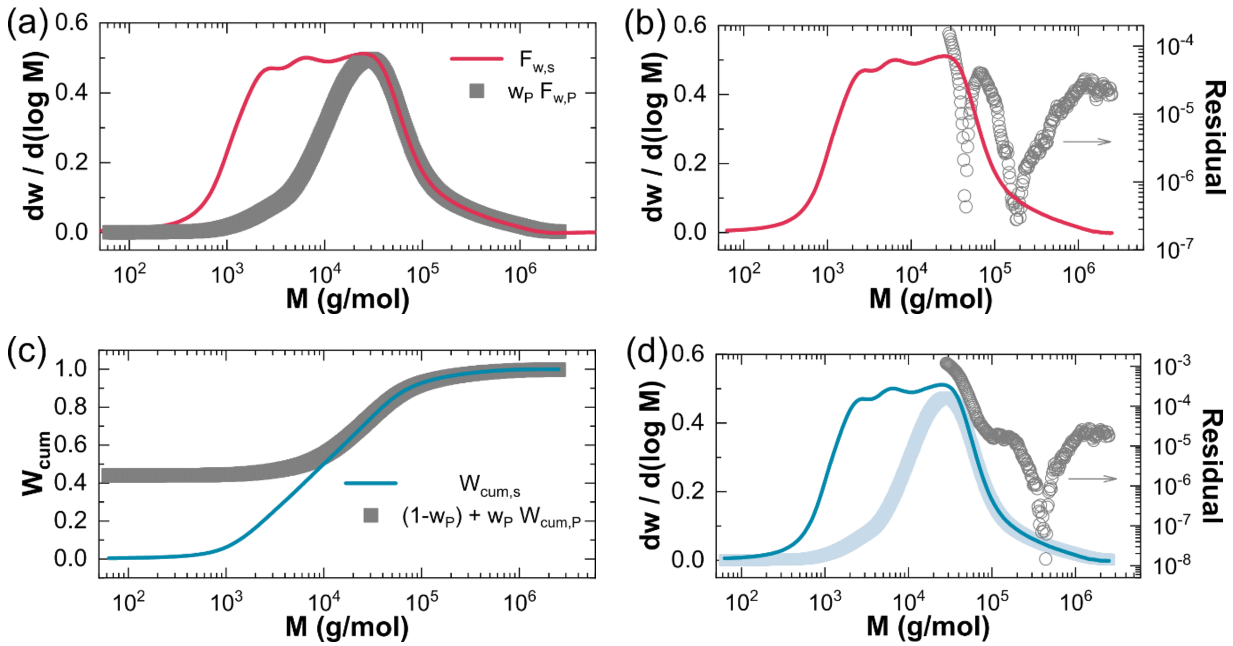

Figure S3. Demonstration of results from fitting (a) $F_{w,s}$ and (c) $W_{cum,s}$. (b) Residuals between $F_{w,s}$ and $w_P\,F_{w,P}$ and (d) the same for $F_{w,s}$ reconstructed from $W_{cum,P}$ (symbols).

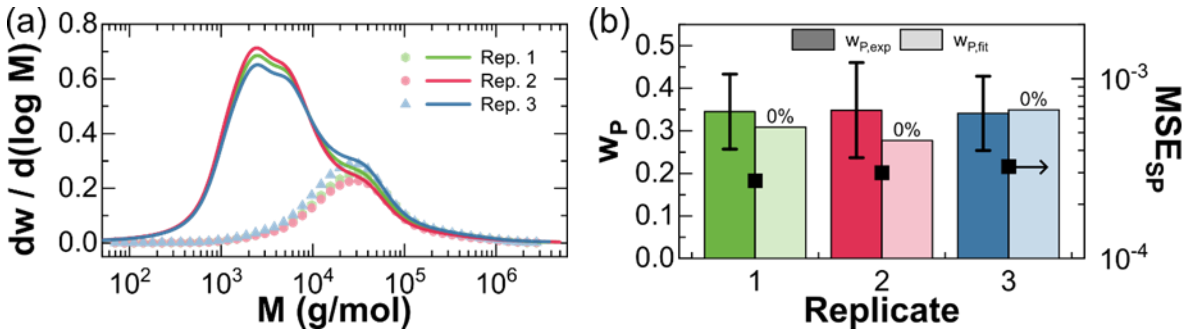

Figure S4. Assessment of method reproducibility for three equivalent surrogate blends of LDPE and HDPE. (a) Measured distributions (line) and fits (symbols). (b) Comparison of $w_{P,exp}$ (with error bars indicating propagated standard deviation) and $w_{P,fit}$, including the resultant MSE_{SP}. $w_{P,fit}$ values have no propagated standard deviation, thus columns are labeled with 0%.

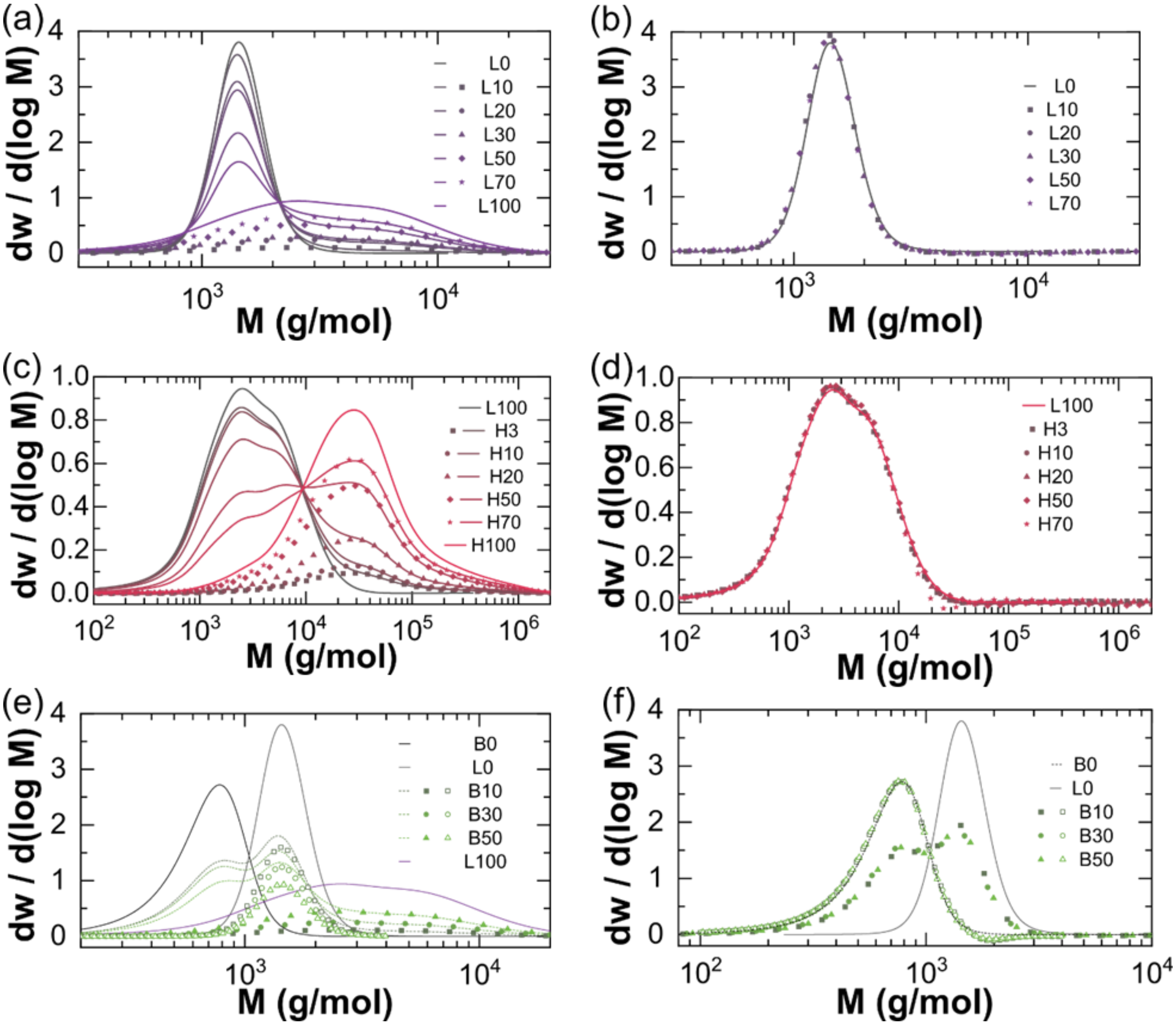

Figure S5. (a, c, e) Measured distributions (lines) and fits (symbols) for the surrogate blends in the L, H, and B series (Table S2), respectively. The open symbols (e) are fits of $F_{w,SP}$ to the L0 component that have been adjusted considering what w_P would be in the entire blend. (b, d, f) $F_{w,SP}$ (symbols) compared to the known component distributions. For (f), $F_{w,SP}$ (open symbols) was generated by removing the intact distributions from both the L100 and L0 components. Filled symbols are the intermediate $F_{w,SP}$ values calculated after the removal of the L100 component.

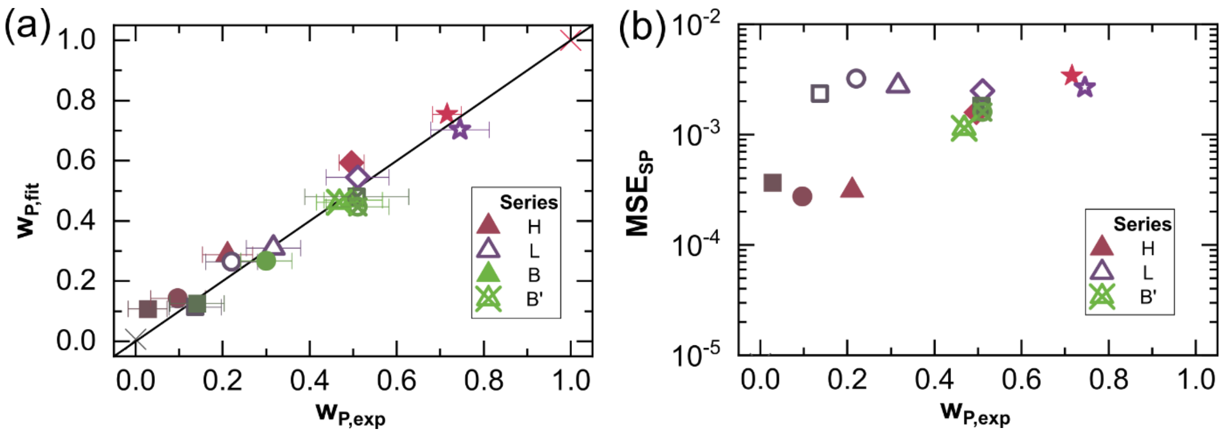

Figure S6. (a) Parity plot of the calculated and experimentally measured w_P and (b) mean-squared error of the calculated solid product distribution for fitting without application of the penalty method.

2.4 Improving the Fit Quality

It is reasonable that a local minimum in the MSE of the fit exists such that a number of polymer chains of some length is predicted to be larger than the measured distribution indicates, thus generating a negative weight fraction of some product species. A set of constraints can be employed to ensure the weight fraction of all product species is positive or zero; these can be written as:

$$F_{w,s} - w_P\, F_{w,P} \geq 0 \tag{S24}$$

We modify our previous solution routine by applying a penalty method, *i.e.*, an unconstrained optimization that achieves a set of constraints by imposing a penalty on the objective function for violations of the constraints. If the fit residual (R_i) is defined as:

$$R_i = F_{w,s}(\log M_i) - w_P F_{w,P}(\log M_i) \tag{S25}$$

and the original sum-of-squares error (SSE) is defined as:

$$SSE = \sum_{M_i=M_{fit}}^{\infty} R_i^2 \tag{S26}$$

A biased sum-of-squares error ($\widehat{SSE}$) can be written as:

$$\widehat{SSE} = \sum_{M_i=M_{fit}}^{\infty} R_i^2 + \frac{\epsilon}{2}\left(1 - \frac{R_i}{|R_i|}\right) R_i^2 \tag{S27}$$

wherein ε is the penalty coefficient (which is independent of M_i). Thus, for a positive residual (within constraint), the penalty term vanishes, and for a negative residual, $\widehat{SSE}$ is incremented by ε additional squared residuals. An optimal value of ε is not known *a priori*; however, the surrogate blends provide the opportunity to both minimize errors in the solid product distribution with respect to the known component distribution and approach the 'true' value of w_P.

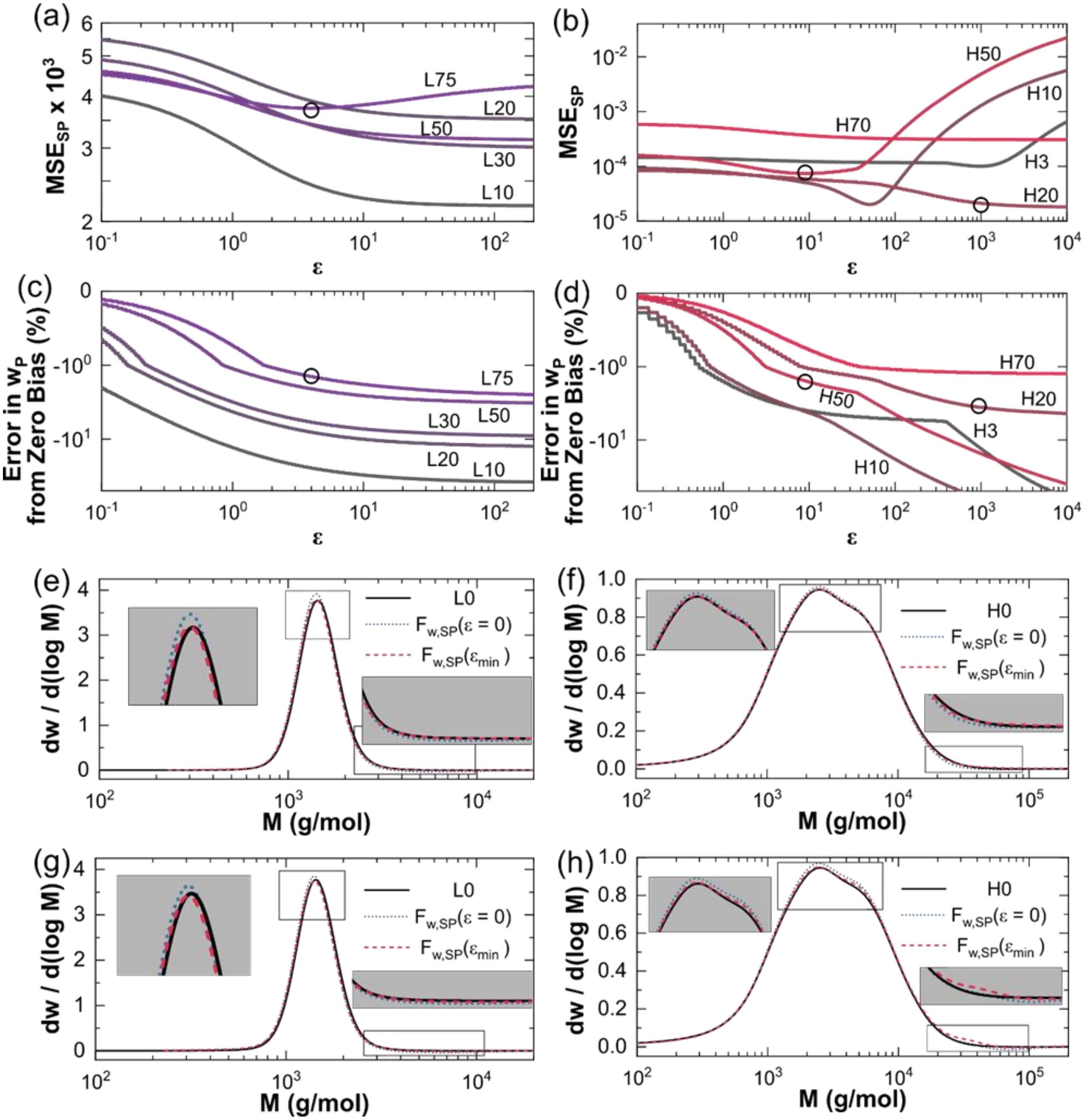

Figure S7. (a) Effect of biasing on the accuracy of the solid product distribution of surrogate blends in the L and H series. (a-b) MSE_{SP} and (c-d) percent error from the $\varepsilon = 0$ case. Circles indicate example points used to generate the distributions in (e-h). Comparison of the solid product distributions from the $\varepsilon = 0$ and ε_{min} cases with the known distribution. Insets show magnified regions of greatest error.

Unfortunately, in real applications of our analysis method, the solid product distribution is an unknown. Thus, the determination of an optimal value for ε must take a different approach, so we use the results for minimization of the solid product distribution as a benchmark for performance. The residual function is being constrained; therefore, the resulting fits typically have the largest residuals near M_{pk}. However, the error in this region is not always desirable to minimize because interference between solid products and the feedstock polymer is likely to vary the goodness of fit at the peak. Hence, our approach to determining ε examines the MSE of the solid products (MSE_{SP}) as a function of ε for a given sample. As shown in **Figure S8a**, the MSE for small penalties increases moderately, reflecting the expected behavior around M_{pk}. When the fit truly becomes poor, MSE is more strongly dependent on ε because the fit diverges from the measured distribution. This dependence occurs over finite ε, because at some point, the penalty term becomes active for only a fixed, small number of points.

Given the behavior of the MSE, our approach for the determination of an optimal penalty coefficient (ε_{opt}) is based on the occurrence of the greatest change in the trajectory (*i.e.*, slope) of MSE with ε. Thus, we define the optimum as:

$$f(\epsilon_{opt}) = \max_{\epsilon}\left\{\frac{d\left[\log\left(\frac{MSE}{MSE(\epsilon = 0)}\right)\right]}{d(\log \epsilon)}\right\} \qquad \textbf{(S28)}$$

that is, the global maximum of the logarithm of the normalized (by the zero-penalty case) MSE as a function of the logarithm of ε. As show in **Figure S8b**, the ε from **(S28)** does not occur at the same value of ε that was determined by the minimum error in the solid product distribution; however, the two ε values are linearly correlated.

The resulting fits and solid product distributions (**Figure S8c-d**) demonstrate an improvement in the method over the unconstrained ($\varepsilon = 0$) case, although some discrepancy between $F_{w,SP}$ and the known L100 distribution occurs near M_{pk}. The summary of errors for the H series (**Figure S8e**) demonstrates that, in all cases, the fit value of w_P is closest to the experimental value for the penalty method using ε_{opt}. Additionally, the error between w_P and $w_{P,exp}$ is inversely proportional to $w_{P,exp}$. Although it was not expected to decrease MSE_{SP} below the pre-determined minimum, MSE_{SP} for the ε_{opt} case was sometimes greater than for $\varepsilon = 0$.

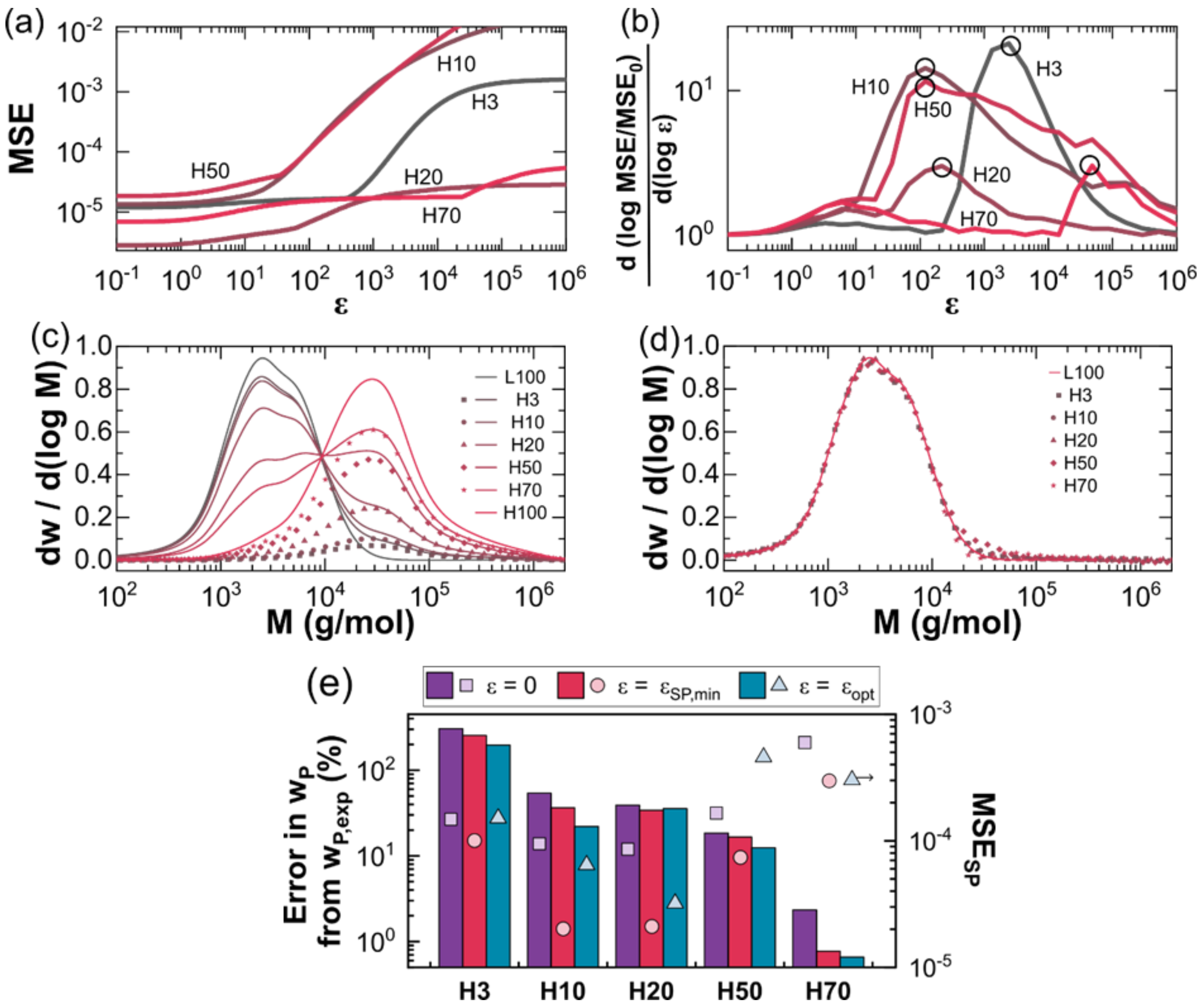

Figure S8. Results for the penalty method. (a) MSE of the fit and (b) differential normalized MSE as a function of ε. Circles in (b) indicate the location of ε_{opt}. (c) Measured blend molar mass distributions (lines) and fits (symbols) and (d) solid product distributions for the H series utilizing values of ε_{opt}. (e) Summary of different penalty coefficient approaches, *i.e.*, no penalty, ε from the minimum in MSE_{SP}, and ε_{opt}. Bars correspond to standard error from the experimentally measured $w_{P,exp}$, and symbols represent MSE_{SP}.

In cases for which a small violation of the imposed constraint cannot be avoided, an additional step can be employed to reduce the subtraction error in the solid product distribution. We have noticed that this step is typically required only in the event of a slightly misshapen distribution in the region corresponding to the intact fraction, such that the overall value of w_P is a good estimate, but $F_{w,SP}$ fluctuates around zero in this region (at a high magnitude because of the large value of

M). Thus, our approach corrects $F_{w,SP}$ by selecting the first (set of) peak(s) by cutting off the data beyond the first point $F_{w,SP} < 0$ within some tolerance (*e.g.*, 5%). The new distribution is then generated by renormalizing the selected peak(s) by $\int F_{w,SP}\, d(\log M)$.

2.5 Deconvolution of Molar Mass Distributions

Deconvolution of molar mass distribution populations was performed by first fitting log-Normal distributions in the form:

$$g(M) = \frac{1}{\sigma\sqrt{2\pi}} \exp\left[-\frac{1}{2}\frac{(\ln M - \ln\mu)^2}{\sigma^2}\right] \quad \textbf{(S29)}$$

wherein μ and σ are the mean and standard deviation of the distribution, respectively. For a set of N populations, the overall distribution can be written as:

$$G(\log M) = \sum_{i=1}^{N} m_i\, g_i(M) = \sum_{i=1}^{N} \frac{m_i}{\sigma_i\sqrt{2\pi}} \exp\left[-\frac{1}{2}\frac{(\ln M - \ln\mu_i)^2}{\sigma_i^2}\right] \quad \textbf{(S30)}$$

wherein m_i, the weighting factor, μ_i, and σ_i exist for each population. The properties of the populations can be written as:

$$M_n = \mu \exp\left(-\frac{\sigma^2}{2}\right) \quad \textbf{(S31)}$$

$$M_w = \mu \exp\left(\frac{\sigma^2}{2}\right) \quad \textbf{(S32)}$$

$$M_z = \mu \exp\left(\frac{3\sigma^2}{2}\right) \quad \textbf{(S33)}$$

$$Đ = \exp(\sigma^2) \quad \textbf{(S34)}$$

To achieve a meaningful fit to molar mass distributions, a constrained minimization is performed such that the sum-of-squared residuals is minimized subject to the following constraints:

1. all parameters are positive,
2. the populations sum to the whole measured distribution, *i.e.*,

$$\int_{-\infty}^{\infty} G(\log M) d(\log M) = \int_{-\infty}^{\infty} \frac{dw}{d(\log M)} d(\log M) \equiv 1 \qquad \textbf{(S35)}$$

$$\therefore \sum_{i=1}^{N} m_i = \ln 10 \qquad \textbf{(S36)}$$

3. the peak center is within a small margin of the guess, specifically:

$$10^{-\lambda} \cdot \mu_{i,guess} \leq \mu_i \leq 10^{\lambda} \cdot \mu_{i,guess} \qquad \textbf{(S37)}$$

4. peaks are of narrow dispersity, *i.e.*,

$$Đ < Đ_{max} \qquad \textbf{(S38)}$$

$$\therefore \sigma_i \leq \sqrt{\ln Đ_{max}} \qquad \textbf{(S39)}$$

The constrained minimization problem defined by **(S30, S35-S39)** is solved using a sequential quadratic programming (SQP) algorithm *via* MATLAB's *fmincon* function. The input to the function requires only an initial guess of the parameter set [m_i, μ_i, σ_i] given N and the measured distribution. The probability that a local minimum is found is high because of the size of the problem. To mitigate this issue, we apply an annealing step to the solution such that subsequent minimizations are performed using small, random perturbations around the initial solution ($[y]_0$) as guesses ($[y]_{guess,k}$), defined as:

$$[y]_{guess,k} = [y]_0(1 + \theta\mathbb{R}) \quad \textbf{(S40)}$$

wherein θ is a perturbation constant, and the random number is given by $\mathbb{R} \subset [-1\,,1]$. The optimal solution, which returns the smallest mean-squared error among the initial solution and 20 random perturbations, is chosen.

2.6 Additional GPC Results

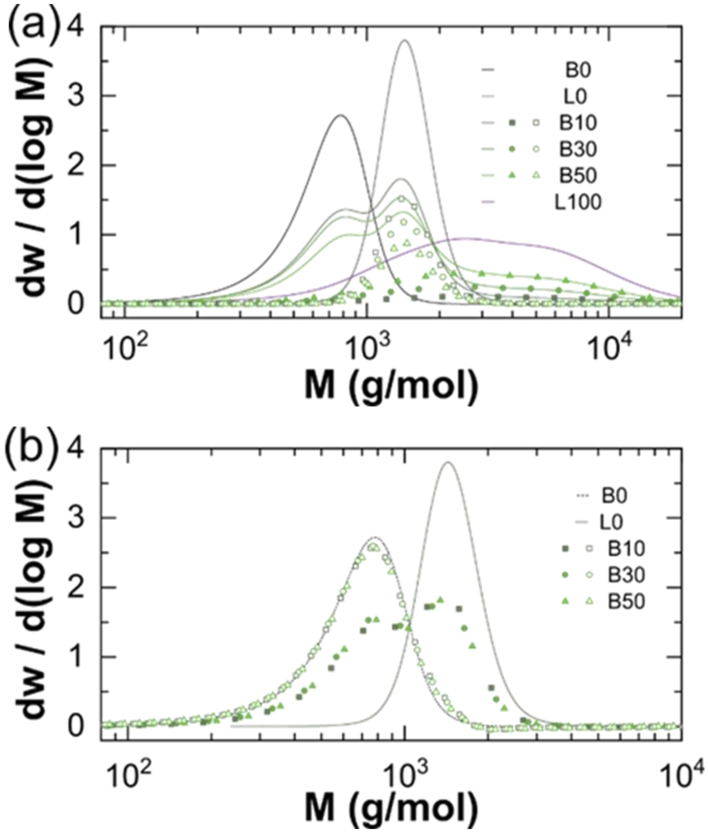

Figure S9. (a) Measured distributions (lines) and fits (symbols) for the surrogate blends B-series utilizing values of ε_{opt}. Closed symbols represent fits of the L100 component, and open symbols are for fits of the L0 component that have been adjusted considering what w_P would be in the entire blend. (b) $F_{w,SP}$ (symbols) compared to the known component distributions. Closed and open symbols are for $F_{w,SP}$ generated by removing the intact distributions from the L100 and L0 components, respectively.

2.7 Log-Normal Distributions of Molar Mass

A series of log-Normal distributions are used to describe the entire distribution of deconstruction products (**Figure S10**). This approach allows more detailed quantification than calculating average molar masses (*i.e.*, moments of the entire distribution) by quantifying the mass of narrow dispersity components. The log-Normal distributions for hydrocracked LDPE after 4 h and pyrolyzed LDPE at 0% mass loss are shown in **Figures S10a** and **S10b**, respectively. The distributions are applied to all solids and are located in **Figures 4f, 5f,** and **6c**.

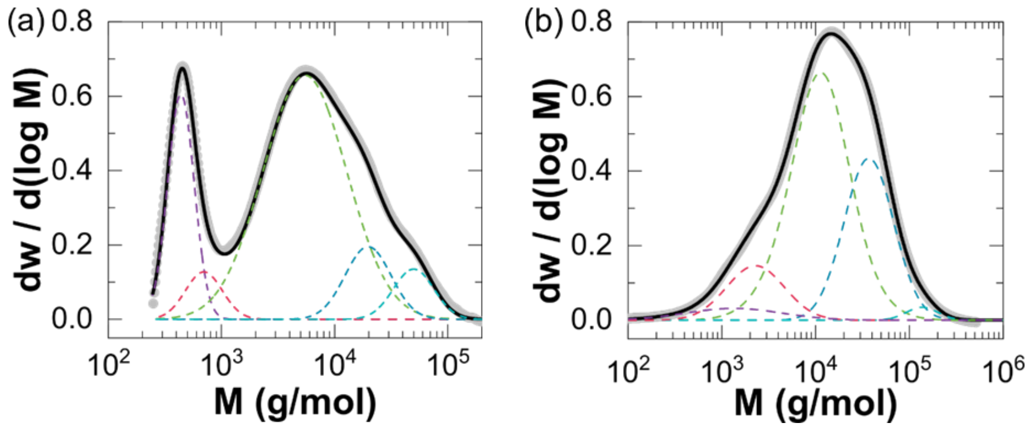

Figure S10. Schematic example deconvoluted solid product distributions from (a) hydrocracking (4 h) and (b) pyrolysis (425 °C, 0% loss). Grey symbols are the measured distributions, and black lines are the sum of components distributions (dashed lines).

2.8 Self-Seeding and Annealing (SSA) Methodology

Determining the proper seeding temperatures for the SSA methodology first requires identification for three regimes of self-nucleation.[13] All thermal history needs to be removed prior to annealing, which is accomplished by using a 5 min isothermal hold at 150 °C, above the equilibrium melting temperature (T_m^0) of PE.[14] Seeding temperatures (T_s) are chosen between the melting region of the polymer sample and its T_m^0, and isothermal hold times (t_s) were always 5 min. **Figure S11**

describes the thermal protocol for these experiments and highlights the cooling and heating curves of interest.

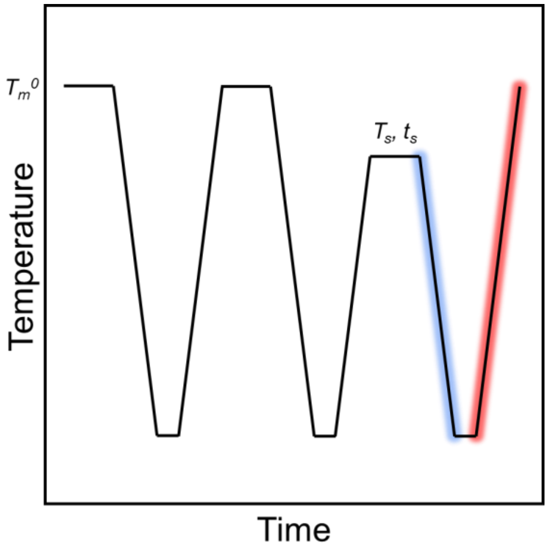

Figure S11. DSC experimental design schematic used to determine the domain of self-seeding and annealing. After annealing at T_s, the cooling curve (highlighted in blue) and heating curve (highlighted in red) are recorded for the crystallization and melting temperatures, respectively.

The blue and red highlighted line segments in **Figure S11** provide the information required to determine the three domains of self-seeding and annealing. When the crystallization peak after an isotherm at or above T_m^0 is referenced after any annealing step at T_s, and when the crystallization peak temperature remains unchanged compared to the peak after T_m^0, then the T_s is within Domain 1 (D1), see **Figure S12**. Once the crystallization peak shifts to higher temperatures, the T_s is within Domain 2 (D2), which describes an increase in nucleation density. Once a T_s change leads to an additional high temperature melting peak, indicating a partially molten sample, Domain 3 (D3) is reached. **Figure S12** shows the heating and cooling curves obtained from annealing at multiple

seeding temperatures, along with the associated peak crystallization and melting points, with dotted lines separating the three Domains (D1, D2, D3).

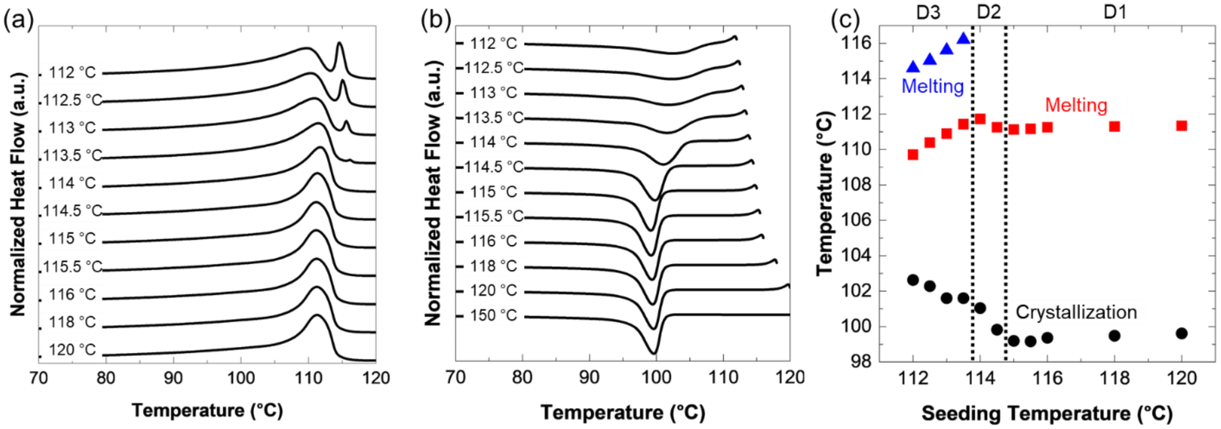

Figure S12. Determining the three domains of self-seeding and annealing of LDPE (a) heating curves after annealing at the T_s labeled on the left-side of the corresponding trace. (b) Cooling curves after annealing at the T_s labeled on the left-side of the corresponding trace. (c) Peaking crystallization temperature (black, circles), peak melting temperature (red, squares), and secondary melting temperature (blue, triangles), plotted versus the seeding temperature, T_s. The three Domains are separated by vertical dotted lines, with the associated labels at the top of the graph.

The first seeding temperature to start the SSA protocol (T_{s1}) is chosen as the lowest temperature in Domain 2, which is 114 °C (**Figure S12c**). The SSA protocol is schematically described in **Figure S13**.

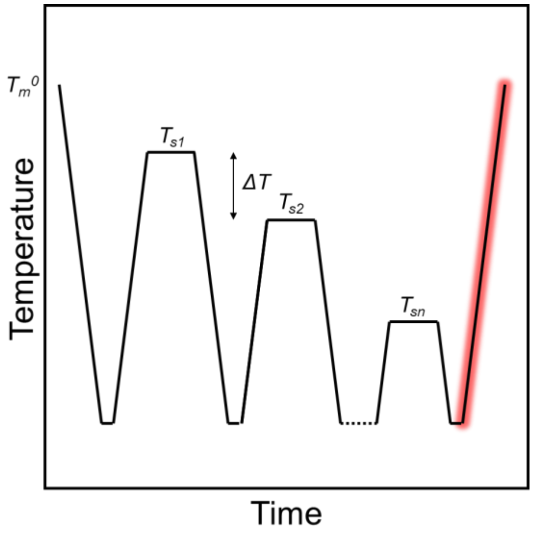

Figure S13. DSC experimental design schematic describing the SSA protocol. Seeding temperatures start at T_{s1}, followed by T_{s2}, and up to as many annealing steps T_{sn}, as desired. The heating curve (red, highlighted) following annealing temperature, T_{sn}, has a corresponding melting event for the sample that contains information about the branching architecture.

The SSA protocol has no minimum or maximum number of required annealing steps; however, as more annealing steps are applied, more precise information of the branching architecture is accessible. For this study, 15 total annealing steps were applied to each sample as the resulting melting curves still had easily identifiable peaks. The change in temperature between annealing steps (ΔT) was chosen as 3 °C for similar reasons as the 15 total annealing steps (well-resolved peaks in the melting curves). ΔT < 3 °C created significant peak overlap, and ΔT > 3 °C did not provide as much information. The total number of annealing steps and ΔT that produce melting traces with easily identifiable peaks are influenced by the instrument and sample size; therefore, the same instrument and relatively similar sample sizes (4-5 mg) were used for all samples.

2.9 Blend Compositions for DSC Methodology

The composition, in weight fraction, for the surrogate blends used in the DSC experiments (**Figure 3**) are listed in **Table S3**. Only $LDPE_{low}$ and PW1000 are used in the DSC experiments as their melting points overlapped the most.

Table S3. Composition (w/w) of the surrogate blends used in DSC experiments.

	**$LDPE_{low}$**	**PW1000**
**100:0**	1.00	0.00
**75:25**	0.74	0.26
**50:50**	0.51	0.49
**30:70**	0.32	0.68
**20:80**	0.20	0.80
**10:90**	0.10	0.90
**0:100**	0.00	1.00

2.10 Fits to SSA Melting Curves

The heating curves after the applied SSA protocol are shown for all samples in this study (**Figure S14-S17**). The curves were baseline corrected with the sigmoidal baseline feature in the Trios software (TA Instruments), and the Gaussian fitting only occurs over the melting region that contained well-resolved peaks (generally, 70-120 °C). The melting curves were fit with a series of 15 Gaussian curves because of the 15 applied annealing steps, and the curves were defined by:

$$g(T) = \frac{1}{\sigma\sqrt{2\pi}} \exp\left[-\frac{1}{2}\frac{\left(T - T_{pk}\right)^2}{\sigma^2}\right] \quad \textbf{(S40)}$$

wherein T_{pk} is the peak melting temperature, and σ^2 is the variance. The peak temperatures and areas of the individual Gaussian curves were used to calculate the methylene sequence length and relative area of each lamellar thickness population.

The high linearity of PW1000 was indicated by only three strong melting peaks after fractionation, whereas $LDPE_{low}$ had defined melting peaks that spanned over 40 °C, which highlighted the more complex branching architecture of $LDPE_{low}$. As surrogate blends, the more branched $LDPE_{low}$ was more representative of reacted product species than the more linear PW1000. An increase in the amount of $LDPE_{low}$ accompanied an expected increase in the melting range and resolution of melting peaks at lower temperatures. Because the SSA methodology already fractionates the crystals *via* methylene sequence lengths, phase mixing or separation of $LDPE_{low}$ and PW1000 does not affect the analysis, as all methylene sequence lengths will be taken into account.

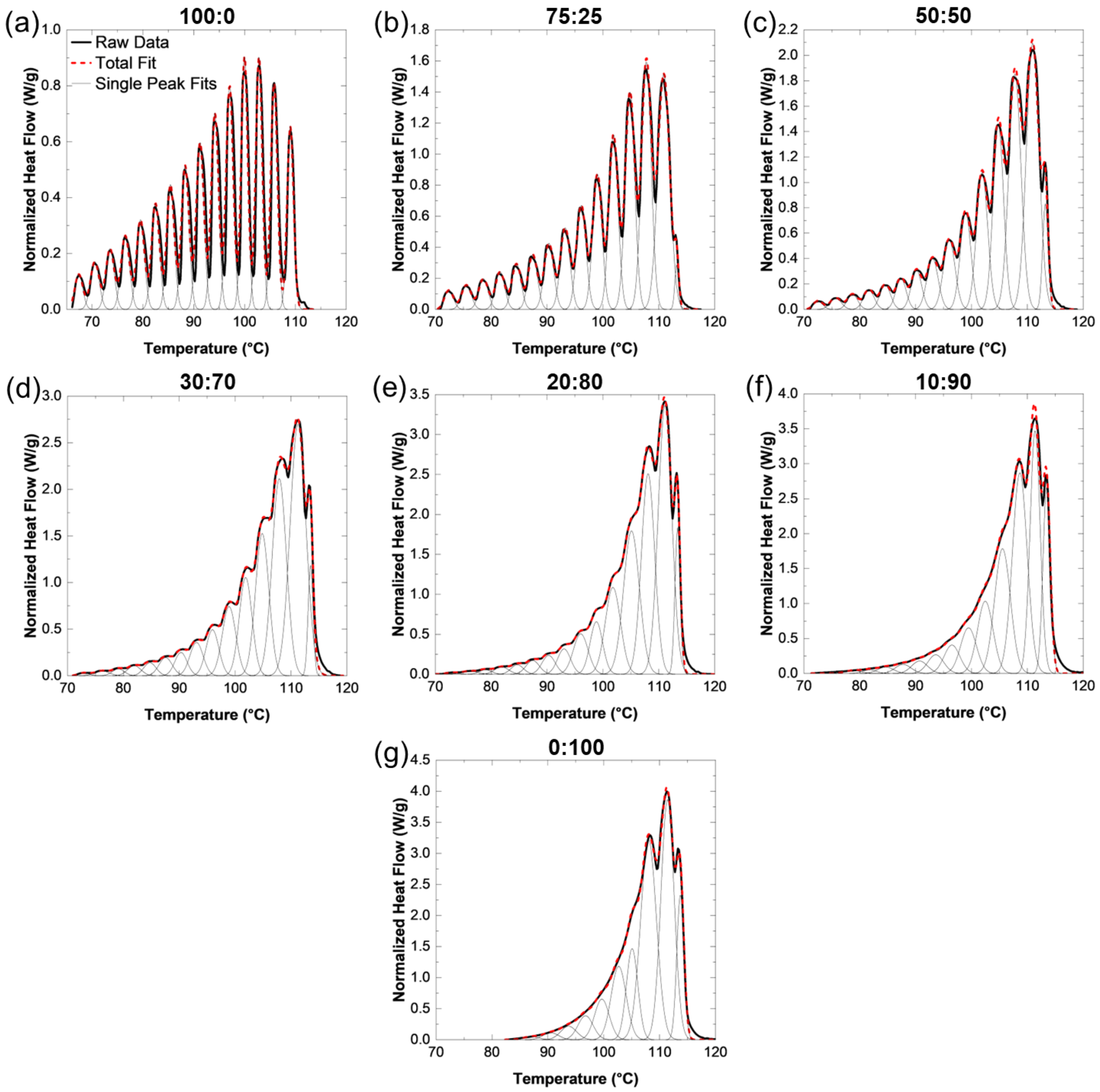

Figure S14. Melting curves of the surrogate blends after the SSA thermal protocol. (a-g) Raw data heating curves (black, solid line) after baseline correction, individual single Gaussian fits (gray, solid line), and total summed fit of all Gaussians (red, dotted line) are shown for each sample. The blend compositions are provided at the top of the graph, and exact fractions are listed in Table S3.

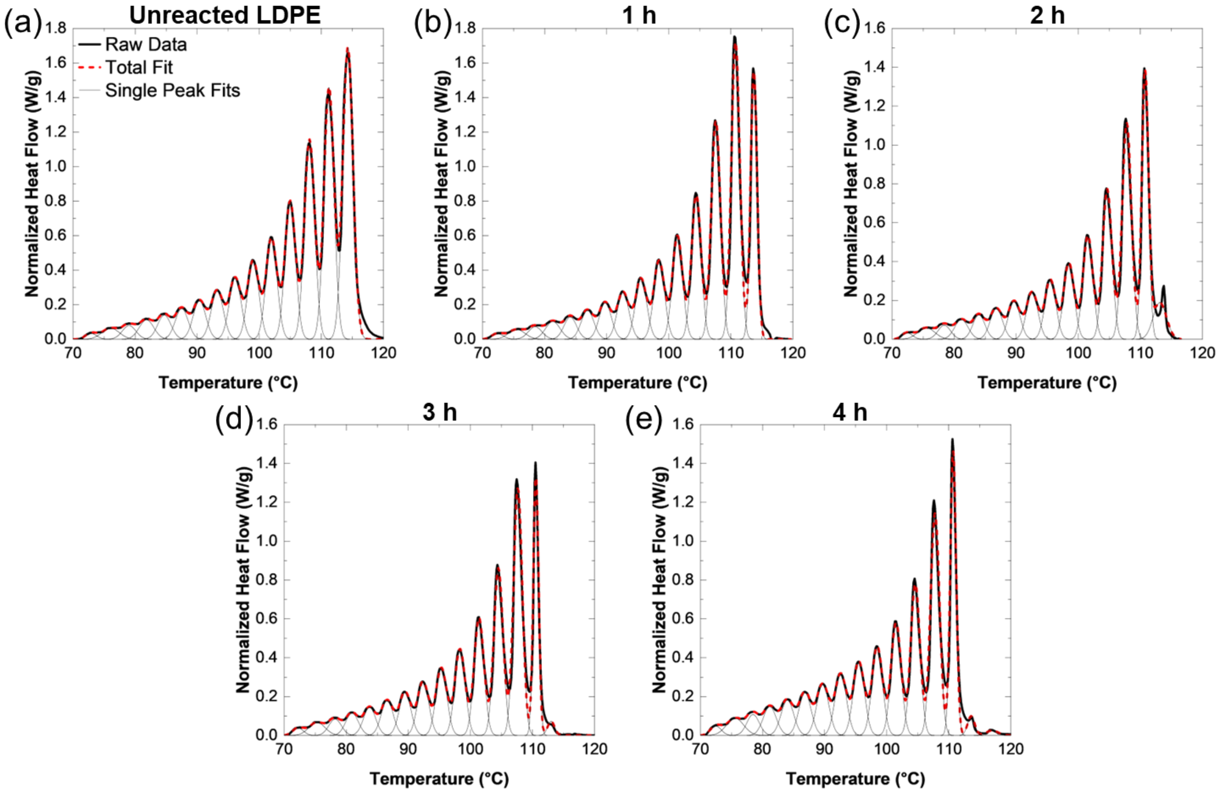

Figure S15. Melting curves of the solids after hydrocracking upon implementation of the SSA thermal protocol. (a-e) Raw data heating curves (black, solid line) after baseline correction, individual single Gaussian fits (gray, solid line), and total summed fit of all Gaussians (red, dotted line) are shown for each sample. The hydrocracking reaction times are provided at the top of each graph.

Figure S15 shows the melting curves and Gaussian fits for all hydrocracking solids from the kinetic series found in **Figure 4**. As hydrocracking time was increased, the overall melting range for the solids remained similar, yet the intensity of the highest temperature melting peak at ~115 °C decreased. Even after 4 h, this peak was still observable, which highlights that unreacted LDPE remained in the solids. There was no significant increase in the melting intensities or trend at lower temperatures (70-90 °C).

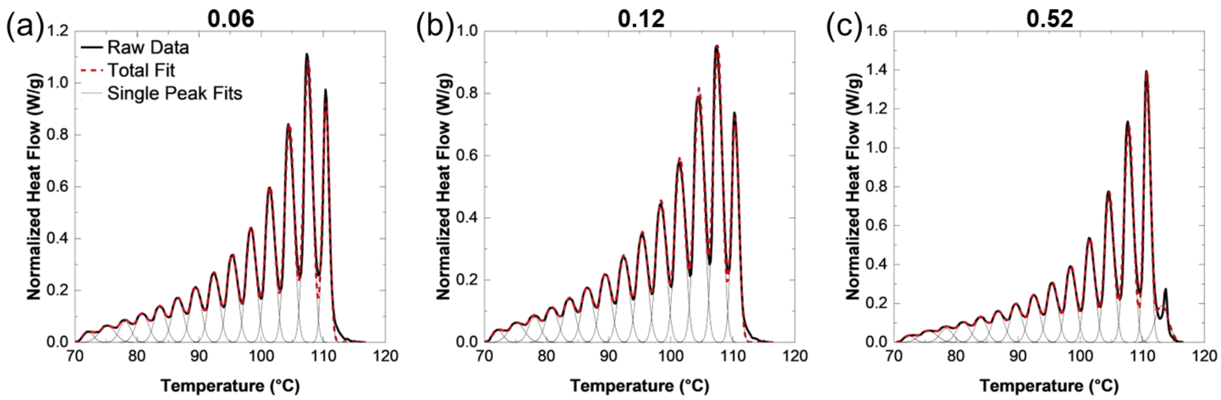

Figure S16. Melting curves of the solids of hydrocracking after the SSA thermal protocol. (a-c) Raw data heating curves (black, solid line) after baseline correction, individual single Gaussian fits (gray, solid line), and total summed fit of all Gaussians (red, dotted line) are shown for each sample. The metal-acid balances of the hydrocracking catalyst are provided at the top of each graph.

Figure S16 shows the melting curves and Gaussian fits for all hydrocracking solids from the metal-acid balance (MAB) series found in **Figure 5**. In contrast to the kinetic series, the melting peak intensities and temperatures varied more with MAB.

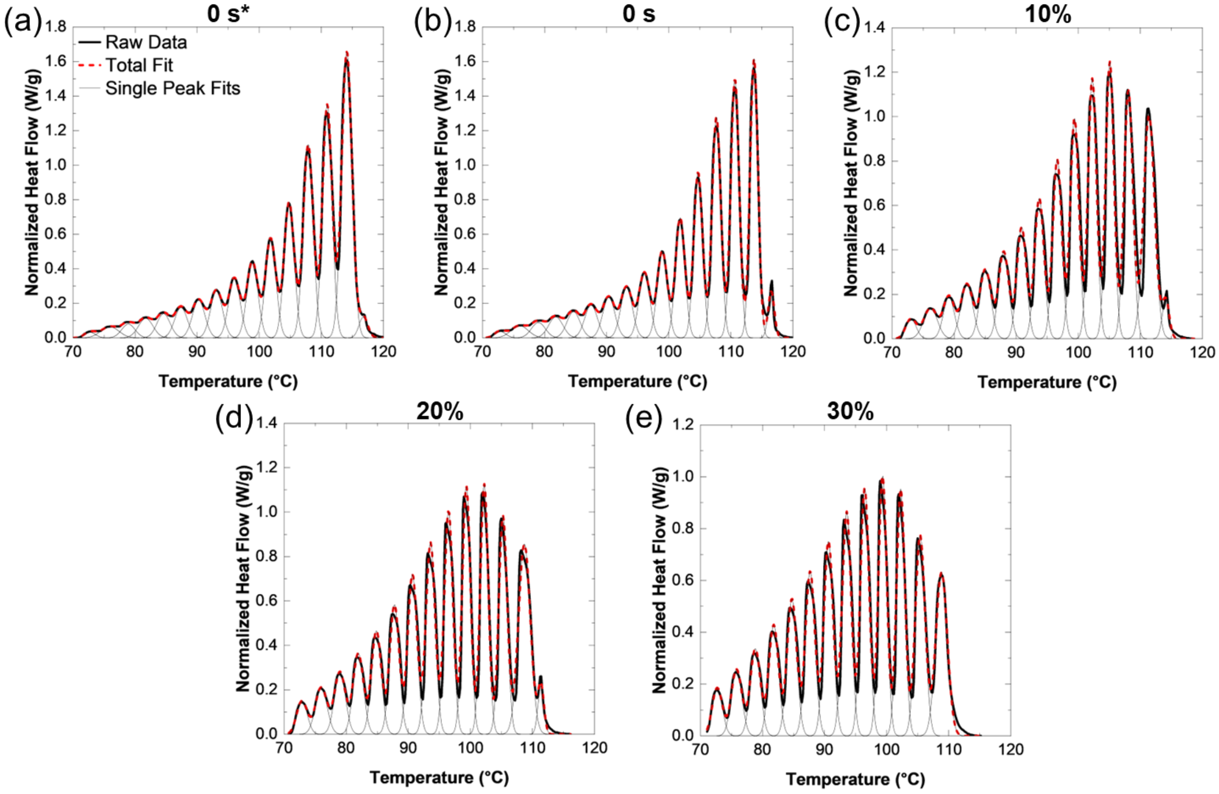

Figure S17. Melting curves of the solids from pyrolysis after the SSA thermal protocol. (a) Melting curve of the sample pyrolyzed for 0 s at 400 °C. (b) Melting curve of the sample pyrolyzed for 0 s at 425 °C. (c-e) Raw data heating curves (black, solid line) after baseline correction, individual single Gaussian fits (gray, solid line), and total summed fit of all Gaussians (red, dotted line) are shown for each sample with the mass loss after pyrolysis provided at the top of each graph.

Figure S17 shows the melting curves and Gaussian fits for solids from the pyrolysis series in **Figure 6**. The pyrolyzed samples showed a more significant increase in intensity of the lower melting temperature peaks, which was expected given the bulk nature of the reaction (*i.e.*, random chain scission across the whole backbone).

2.11 Thermogravimetric Analysis

Figure S18 shows the mass loss over time of LDPE from isothermal pyrolysis at 425 °C. The residual mass after pyrolysis was assumed to be all solids, which was reasonable given that the molar mass distributions from **Figure 6** suggested very little low-molar-mass species (< 500 g/mol).

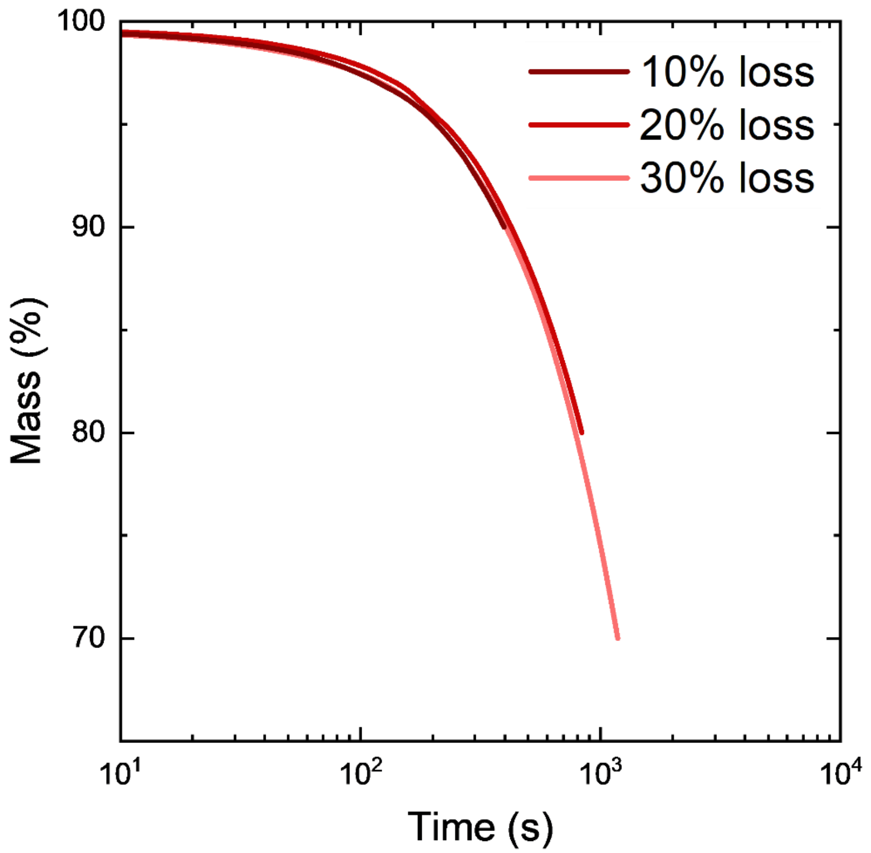

Figure S18. Mass loss versus time during isothermal pyrolysis of LDPE at 425 °C

Samples pyrolyzed for 0 s at 400 °C and 425 °C (**Figure 6** and **Figure S19**) had mass losses less than 0.5%. The samples did not fully reach the desired pyrolysis temperature due to the relatively

large size (15 mg), low thermal conductivity of the samples, and 50 °C/min heating rate. However, the pyrolysis temperature was assumed to have been reached within seconds of the isothermal holds, thus not significantly impacting the results. Slight variations between samples can be seen as the lines in **Figure S18** do not perfectly overlap.

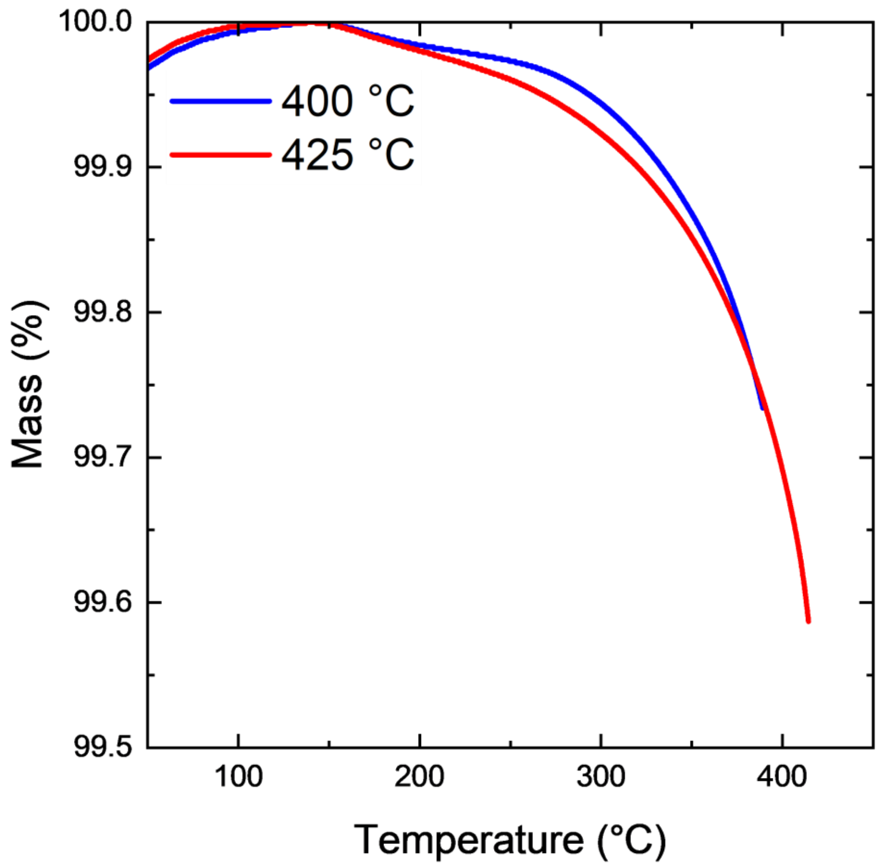

Figure S19. Mass loss versus temperature for samples pyrolyzed up to 400 °C (blue) and 425 °C (red). Samples were immediately cooled upon reaching the pyrolysis temperature, so no isotherms were applied. Mass loss was less than 0.5% for each sample.

LDPE lost less than 0.5% of its original mass prior to reaching the pyrolysis temperatures, yet **Figure 6** highlights a significant shift in molar mass of the LDPE for these samples. The slight

increase in mass in the beginning of the measurement was attributed to the settling TGA pans and not mass gain of the LDPE.

2.12 Viscosity

After the frequency sweep, the rheological data were transformed using the Cox-Merz rule,[15] described as:

$$|\eta^*(\omega)| = \eta(\dot{\gamma}) \quad \textbf{(S41)}$$

wherein, $\eta^*(\omega)$ is the complex viscosity as a function of angular frequency (ω), and $\eta(\dot{\gamma})$ is the viscosity as a function of shear rate ($\dot{\gamma}$). The zero-shear viscosity (η_0) was then calculated by fitting the complex viscosity *vs.* shear-rate data (**Fig. S20**) according to the Cross model,[16] described as:

$$\frac{\eta(\dot{\gamma}) - \eta_\infty}{\eta_0 - \eta_\infty} = \frac{1}{1 + (c\dot{\gamma})^p} \quad \textbf{(S42)}$$

wherein, η_∞ is viscosity at infinite-shear, c is the Cross constant, and p is the Cross exponent. η_0 was 127 Pa s (250 °C) for neat LDPE. A constant viscosity was found for $LDPE_{low}$, but the same shear-rate range could not be measured due to instrument resolution, so η_0 was calculated as the average value across the shear rates measured and was determined to be 2 x 10^{-2} Pa s (at 250 °C).

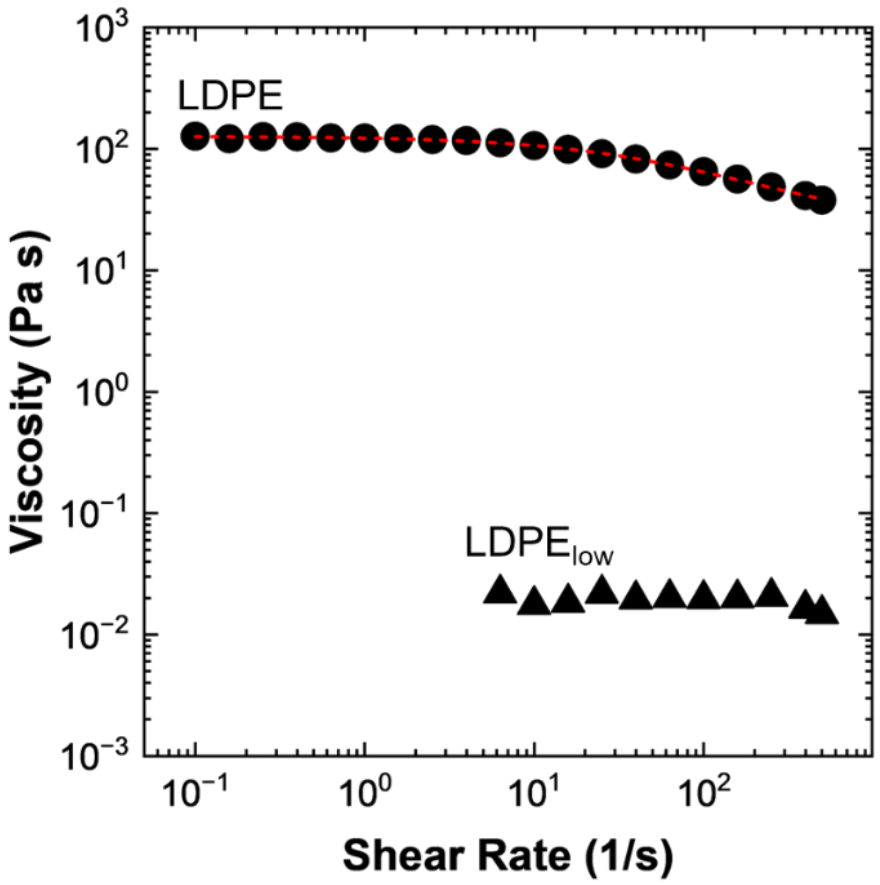

Figure S20. Viscosity as a function of shear rate for LDPE (circles), with its Cross model fit (red line), and for LDPE$_{low}$ (triangles).

2.13 Additional Catalyst and Reaction Details

Exposed Pt metal densities were measured using pulse CO chemisorption, with metal densities spanning 2-15 μmol/g_{cat} for Pt loadings ranging from 0.1 - 1.0 wt%. These exposed metal densities yielded dispersions of 0.25-0.39, which estimated the average Pt nanoparticle diameters to span 1.1-1.8 nm across the catalyst series.[1] This relatively narrow particle size range is unable to explain the reactivity differences and changes to the molar mass distributions.

Table S4. Relevant catalyst compositions, reaction times, and solids conversions for the hydrocracking of LDPE used in this work.

Catalyst[a]	MAB	Reaction Time	Solids Yield
		h	%
0.5Pt-15WZr	0.52	1	72
0.5Pt-15WZr	0.52	2	57
0.5Pt-15WZr	0.52	3	46
0.5Pt-15WZr	0.52	4	17
0.1Pt-15WZr	0.06	2	67
0.1Pt-25WZr	0.12	2	54

[a]Catalyst compositions are in the format: *x*Pt-*y*WZr, wherein *y* corresponds to the weight percent loading of WZr on the ZrO_2 support, and *x* corresponds to the weight percent loading of Pt on the *y*WZr support. More details with respect to catalyst synthesis and characterization can be found in Vance, *et al.*[1]

References

(1) Vance, B. C.; Kots, P. A.; Wang, C.; Hinton, Z. R.; Quinn, C. M.; Epps, T. H., III; Korley, L. T. J.; Vlachos, D. G. Single pot catalyst strategy to branched products via adhesive isomerization and hydrocracking of polyethylene over platinum tungstated zirconia. *Applied Catalysis B: Environmental* **2021**, *299*, 120483.

(2) Standard Test Method for Determining Molecular Weight Distribution and Molecular Weight Averages of Polyolefins by High Temperature Gel Permeation Chromatography 1.

(3) Cser, F. About the Lorentz correction used in the interpretation of small angle X-ray scattering data of semicrystalline polymers. *Journal of Applied Polymer Science* **2001**, *80* (12), 2300-2308.

(4) Monnier, X.; Napolitano, S.; Cangialosi, D. Direct observation of desorption of a melt of long polymer chains. *Nature Communications* **2020**, *11* (1), 4354.

(5) Napolitano, S. Irreversible adsorption of polymer melts and nanoconfinement effects. *Soft Matter* **2020**, *16* (23), 5348-5365.

(6) Gin, P.; Jiang, N.; Liang, C.; Taniguchi, T.; Akgun, B.; Satija, S. K.; Endoh, M. K.; Koga, T. Revealed Architectures of Adsorbed Polymer Chains at Solid-Polymer Melt Interfaces. *Physical Review Letters* **2012**, *109* (26), 265501.

(7) Bailey, E. J.; Griffin, P. J.; Composto, R. J.; Winey, K. I. Characterizing the Areal Density and Desorption Kinetics of Physically Adsorbed Polymer in Polymer Nanocomposite Melts. *Macromolecules* **2020**, *53* (7), 2744-2753.

(8) Bailey, E. J.; Winey, K. I. Dynamics of polymer segments, polymer chains, and nanoparticles in polymer nanocomposite melts: A review. *Progress in Polymer Science* **2020**, *105*, 101242.

(9) Bailey, E. J.; Griffin, P. J.; Composto, R. J.; Winey, K. I. Multiscale Dynamics of Small, Attractive Nanoparticles and Entangled Polymers in Polymer Nanocomposites. *Macromolecules* **2019**, *52* (5), 2181-2188.

(10) Wang, K.; Winey, K. I. Vehicular and Core–Shell Nanoparticle Diffusion in Attractive Entangled Polymer Melts. *Macromolecules* **2024**, *57* (14), 6789-6795.

(11) Thees, M. F.; McGuire, J. A.; Roth, C. B. Review and reproducibility of forming adsorbed layers from solvent washing of melt annealed films. *Soft Matter* **2020**, *16* (23), 5366-5387.

(12) Carroll, B.; Bocharova, V.; Carrillo, J.-M. Y.; Kisliuk, A.; Cheng, S.; Yamamoto, U.; Schweizer, K. S.; Sumpter, B. G.; Sokolov, A. P. Diffusion of Sticky Nanoparticles in a Polymer Melt: Crossover from Suppressed to Enhanced Transport. *Macromolecules* **2018**, *51* (6), 2268-2275.

(13) Arnal, M. L.; Balsamo, V.; Ronca, G.; Sánchez, A.; Müller, A. J.; Cañizales, E.; Urbina de Navarro, C. Applications of Successive Self-Nucleation and Annealing (SSA) to Polymer Characterization. *Journal of Thermal Analysis and Calorimetry* **2000**, *59* (1), 451-470.

(14) Wunderlich, B.; Czornyj, G. A Study of Equilibrium Melting of Polyethylene. *Macromolecules* **1977**, *10* (5), 906-913.

(15) Cox, W. P.; Merz, E. H. Correlation of dynamic and steady flow viscosities. *Journal of Polymer Science* **1958**, *28* (118), 619-622.

(16) Cross, M. M. Rheology of non-Newtonian fluids: A new flow equation for pseudoplastic systems. *Journal of Colloid Science* **1965**, *20* (5), 417-437.
